# *mthl1*, a potential *Drosophila* homologue of mammalian adhesion GPCRs, is involved in antitumor reactions to injected oncogenic cells in flies

**DOI:** 10.1073/pnas.2303462120

**Published:** 2023-07-17

**Authors:** Di Chen, Xiao Lan, Xiaoming Huang, Jieqing Huang, Xiaojing Zhou, Jiyong Liu, Jules A. Hoffmann

**Affiliations:** ^a^Sino-French Hoffmann Institute, School of Basic Medical Science, Guangzhou Medical University, Guangzhou 511436, China; ^b^University of Strasbourg Institute for Advanced Study, 67000 Strasbourg, France; ^c^Institute of Molecular and Cellular Biology, CNRS, Insect Models of Innate Immunity (M3I; UPR9022), Strasbourg F-67084, France

**Keywords:** *Drosophila melanogaster*, innate immunity, cancer, *methuselah like 1*, adhesion G-protein-coupled receptor

## Abstract

Injection of OCs into adult male flies induces a strong transcriptomic response in the host flies featuring in particular genes encoding bona fide G coupled proteins, among which the gene for *methuselah* *like 1* is prominent. The injection is followed after a 3-d lag period, by the proliferation of the oncogenic cells. We hypothesized that through the product of *mthl1* the host might control, at least in part, this proliferation as a defense reaction. Through a combination of genetic manipulations of the *mthl1* gene (loss of function and overexpression of *mthl1*), we document that indeed this gene has an antiproliferative effect. Parallel injections of primary embryonic *Drosophila* cells or of various microbes do not exhibit this effect. We further show that *mthl1* controls the expression of a large number of genes coding for chemoreceptors and genes implicated in regulation of development. Of great potential interest is our observation that the expression of the mouse gene coding for the *adhesion G-protein-coupled receptor E1* (*Adgre1,* also known as *F4/80*), a potential mammalian homologue of *mthl1*, is significantly induced by B16-F10 melanoma cell inoculation 3 d postinjection in both the bone marrow and spleen (nests of immature and mature myeloid-derived immune cells), respectively. This observation is compatible with a role of this GPCR in the early response to injected tumor cells in mice.

## Significance

To contribute to decipher the defense mechanisms of flies against tumors in the absence of adaptive immunity, we developed a system of injection of RasV12 oncogenic cells (OCs) into adult males. The host reacted by overexpressing many genes, paramount among which the *methuselah like 1 (mthl1)* gene, a GPCR member of a family initially identified through effects on longevity. Concomitantly we noted a significant proliferation of the OCs in the host, which decreased in *mthl1* KD flies but increased when this gene was overexpressed. Of great potential interest is the observation that the OC-induced expression of the *mthl1* gene is paralleled by the increased expression of *Adgre1*, a putative homologue of *mthl1,* following injection of B16-F10 melanoma cells.

Invertebrates have appeared several hundred millions of years before vertebrates and are estimated to make up some 95% of animal species on earth at present, as compared to 5% for extant vertebrates. They have been confronted since their appearance to an enormous variety of potential microbial aggressors, many of which are still present today. The antimicrobial defense mechanisms of insects have attracted increasing interest over the last decades and many laboratories worldwide have engaged in studies regarding the cellular and molecular basis of their highly efficient defense reactions. Several invertebrate species from various phyletic groups have yielded significant data in this respect. In particular, the genetically tractable *Drosophila* has proved extremely valuable for these studies. Over the years, it has thus become apparent that insects rely only on the innate arm of immune defenses to fight microbes and are devoid of the adaptive arm with its hallmark of immune memory, the basis for vaccination in humans. Significantly, at the end of the 1990s it was understood that innate immunity in vertebrates and invertebrates shared many similarities in terms of receptors of microbial aggressors and subsequent activations of intracellular signaling cascades leading to transcription of genes encoding defense proteins, namely antimicrobial peptides, to oppose the invaders (reviews in ref. 1–9).

It has been known for nearly a century that *Drosophila*, like many invertebrates, can develop tumors and a vast number of investigations have been devoted to the genetic origins of this process (10–13). In contrast, relatively few studies have addressed the question of the recognition of such tumors by the flies and their potential responses to these noninfectious insults (14). We have recently started a series of investigations to decipher the potential recognition mechanisms of tumor cells by host flies and the subsequent molecular and cellular reactions.

To simplify our experimental approach, we based our first studies (15) on a model of injections of OCs into adult males (to avoid as much as possible unwanted interferences with developmental regulations in larvae/pupae and with the process of vitellogenesis in female adults). We noted that the injected cells did not proliferate during the first 3 d postinjections (p.i.). After this apparent lag period, the cell numbers increased markedly. Between day 5 and day 11, a massive proliferation occurred. Until finally, half of the experimental flies had succumbed on day 11. In parallel, we noted that the injections of the OCs induced an early (day 3 p.i.) remarkably strong transcriptomic response in the host flies (cf. ref. 15). Unexpectedly, this transcriptomic response included over one hundred genes encoding chemoreceptors of various families, among which 12 are G-protein-coupled receptor (GPCR) family members. Of note, we confirmed in these experiments that the kinetics of induction and the identities of the induced genes differed markedly from the responses generated by parallel injections of microbes (15).

We have now undertaken a series of functional studies on the roles of the genes induced during the early stages following the injection of oncogenic cells. Here, we present our results obtained while focusing on chemoreceptor genes of the GPCR family induced in our model during the early period up to the massive transcriptomic wave around day 3 p.i. In particular, we report that one of the strongest-induced GPCR gene in our hands belongs to a family of previously described fly genes, which can affect (namely, but not solely) the life span negatively, and was termed by its discoverer S. Benzer for this reason, methuselah (*mth*) (16) in reference to the biblical figure Methuselah reported to have lived up to 996 y (Hebrew bible, Genesis, 5, 27). We now know that *Drosophila mth* belongs to a family of 16 genes (*mth* and *mth like 1* to *15*). Of great interest to our long-term project is the fact that similar genes were discovered in mammals around the same period and shown to play roles as adhesion molecules in various settings (cf. Flybase/NCBI). The sequence similarity is particularly interesting between *mthl1* and *adhesion G-protein-coupled receptor E1* (*Adgre1*). We will center in the following our functional analysis on the *mthl1* gene – whose expression in our experimental flies was one of the strongest after injection of OCs. We will address loss-of-function and gain-of-function mutants in the context of the early host response to the injection of oncogenic cells. We will also include data, which we obtained when following the expression of *Adgre1* after inoculation of cancer cells (melanoma B16-F10) into C57/BL6 mice, which support our idea of similarities between the two models in the present context.

## Results

### The Bona Fide GPCRs Have in Our Model the Widest Distribution among Chemoreceptors Induced by Injected Oncogenic Cells.

We first extended our previous preliminary investigations of the biodistribution of all the chemoreceptors induced in flies on day 3 p.i. This list includes 12 bona fide GPCRs plus other types of chemoreceptors, namely ~30 gustatory receptors (Grs), ~20 olfactory receptors (Ors), ~20 ionotropic receptors (Irs) and ~15 pickpockets (Ppks) (see ref. 15, for the list and comments). We wondered where in the fly body these receptors were expressed and for this, we performed a spatial transcriptomic analysis, which is essentially a technique of in situ sequencing that enables multiplexed mapping of RNAs at nanoscale, subcellular resolution. The feature plot analysis allows a comparison of the expression sites of large numbers of genes in parallel. As a caveat, we note that when the basal expression levels of genes are lower than the detection limit (a cutoff of read depth as 1), their expression sites cannot be demonstrated by feature plot analysis.

**Fig. 1.**
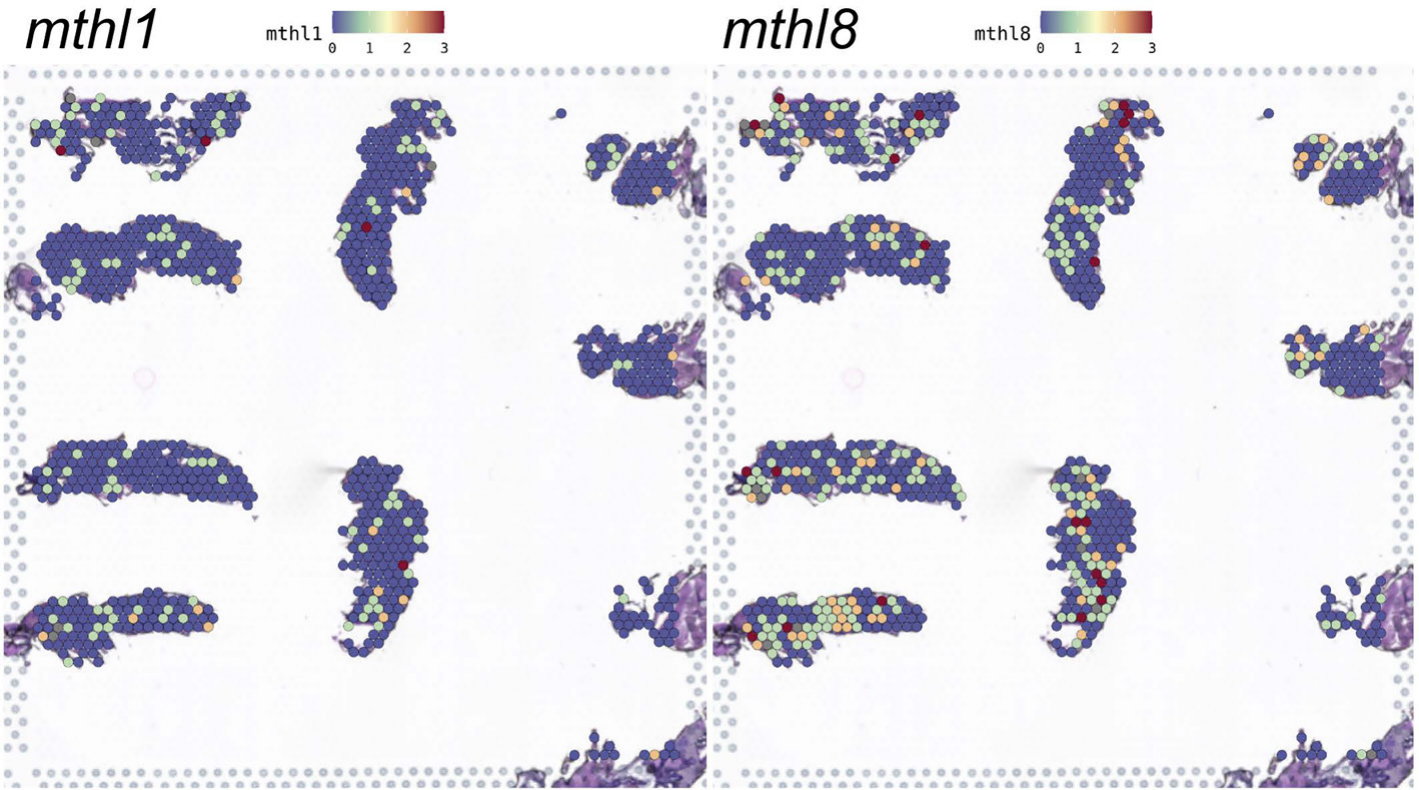
Feature plots indicate a wide random distribution of *mthl1* and *mthl8*. 10 male *W^1118^* wild-type flies receiving OC injection 3 days p.i. were randomly collected and fixed in the embedding box with optimal cutting temperature compound (OCT), then cryosectioned in a thickness of 10 μm/slide for the spatial transcriptomic sequencing. The biodistribution of *mthl1* and *mthl8* was visualized using characteristic feature plot analysis. The relative expression level of different chemoreceptors is indicated by the color of the scale bar (the lowest expression level is set as value 0 in blue and the highest expression level is set as 3 in red).

The result of feature plot analysis shown in Fig. 1 illustrates the expression site of the chemoreceptors from the five families (mentioned above) with basal expression levels above the limit of detection. We noted that overall, the expressions of the members of the bona-fide GPCR chemoreceptors are widespread in all parts/tissues in the flies and that their expression sites are the widest among the 5 chemoreceptor families. As we will discuss below, this is in particular the case for *methuselah like 1* (*mthl1*) and *methuselah like 8* (*mthl8*), which are the widest and most abundantly expressed GPCRs on day 3 p.i. in this study (Fig. 1 and *SI Appendix*, Fig. S1). We found that the majority of expression sites of the Ppks are concentrated in the abdomen (*SI Appendix*, Fig. S1*A*). Similarly, some *Grs*, such as *Gr32a*, *Gr66a,* and *Gr8a* are mostly expressed in the abdomen (*SI Appendix*, Fig. S1*B*). We observed only a few sites of Ors and Irs expression and did not detect any specific patterns of expression of these chemoreceptors (*SI Appendix*, Fig. S1 *C* and *D*).

### Genetic Manipulations (LOF/OE) of *mthl1* Gene Indicate That It Is Involved in Controlling the Proliferation of the Injected OCs.

**Fig. 2.**
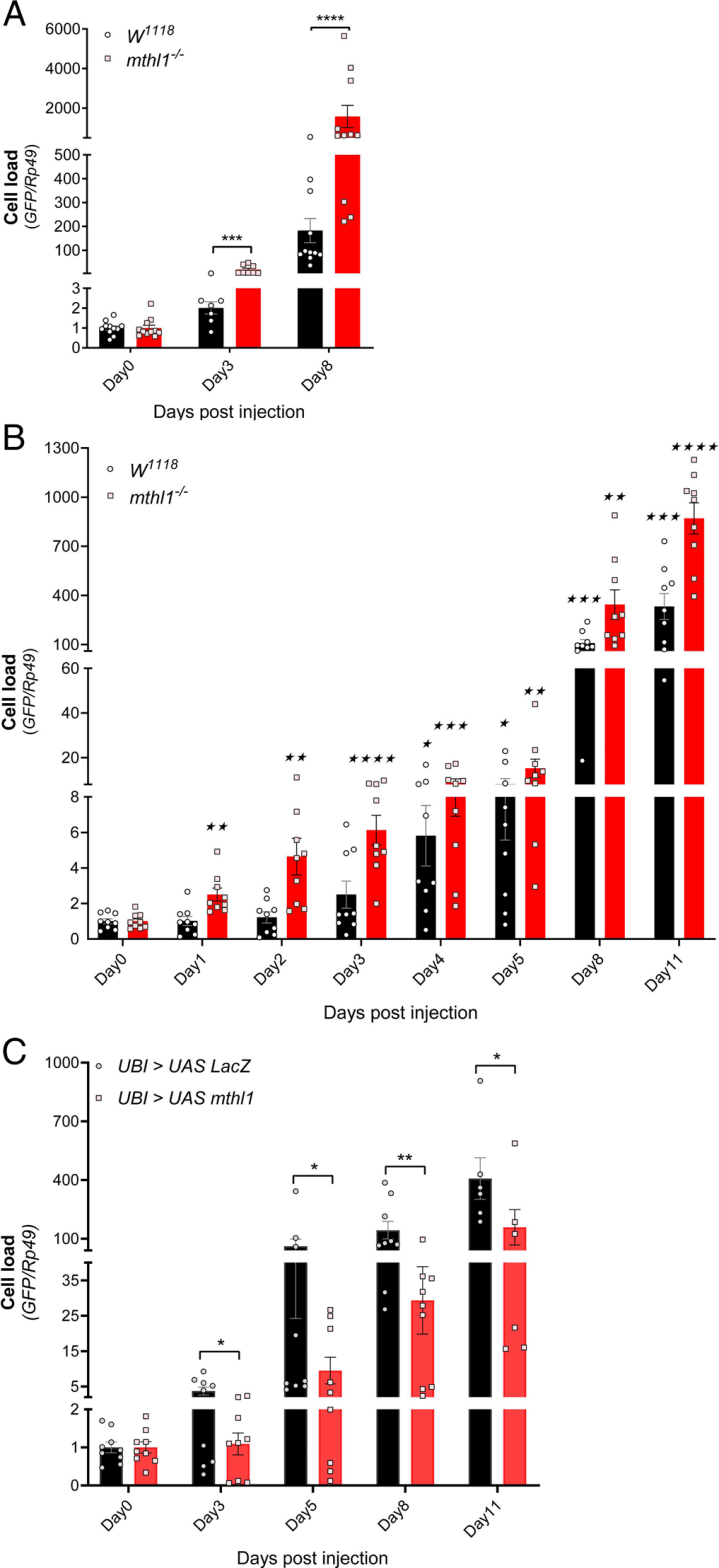
*mthl1* affects the proliferation of injected OCs. (*A*) Cell load of OCs in *W^1118^* wild-type flies and *mthl1* LOF flies at indicated time points after injection. (*B*) Time course record for cell load of OCs in *W^1118^* wild-type flies and *mthl1* LOF flies after injection. (*C*) Cell load of OCs in *UAS-LacZ* flies and *mthl1* overexpression flies at indicated time points after injection. The OCs were injected into flies in a condition of 500 cells/10nl/fly. The total RNAs of flies were then collected at indicated time points after injection and measured by RT-PCR. The cell load of OCs is represented by the relative GFP mRNA expression after normalization to house-keeping gene *Ribosomal protein 49* (*Rp49*). The level of GFP expression on the day of injection (=day 0) is set as 1. One data point represents a pool of six to eight flies for the cell load. The data points are collected from at least three independent experiments. Each experiment includes three to five bioreplicates. Student’s *t* test was used for statistical analysis: (vs. *W^1118^* wild-type flies) *P < 0.05; **P < 0.01; ***P < 0.001; ****P < 0.0001. (vs. value at day 0) ^★^*P* < 0.05; ^★★^*P* < 0.01; ^★★★^*P* < 0.001; ^★★★★^*P* < 0.0001.

A P-element insertion-induced depletion for *mthl1* (Loss of function, LOF hereafter) increased the proliferation of the injected OCs dramatically (~ninefold 3 days p.i., as compared to wild-type flies), (Fig. 2*A*).

To determine the time when the antiproliferative effect of *mthl1* starts to function in response to injected OCs, we performed a time course experiment. As demonstrated in Fig. 2*B*, in *mthl1* LOF flies, the proliferation of OCs started very early (up to 2.5-fold within 24 h p.i.), then increased considerably from day 2 to day 5 (from 4- to 16-fold) and became exponential thereafter (400-fold at day 8 p.i.). Since the doubling time for dish-culture OCs is 13.5 h, we infer that the OCs start to proliferate right after the injection. In contrast, the proliferation of the OCs only started at day 4 p.i. in wild-type flies.

Conversely, flies in which the *mthl1* gene was overexpressed, showed a strongly reduced proliferation of the oncogenic cells (around sixfold on day 5 p.i., as compared to wild-type flies).

### *mthl1* Is Induced by Injected OCs in *Drosophila* and Not by Injection of Embryonic Cells (ECs) or of Injection of Microbes.

**Fig. 3.**
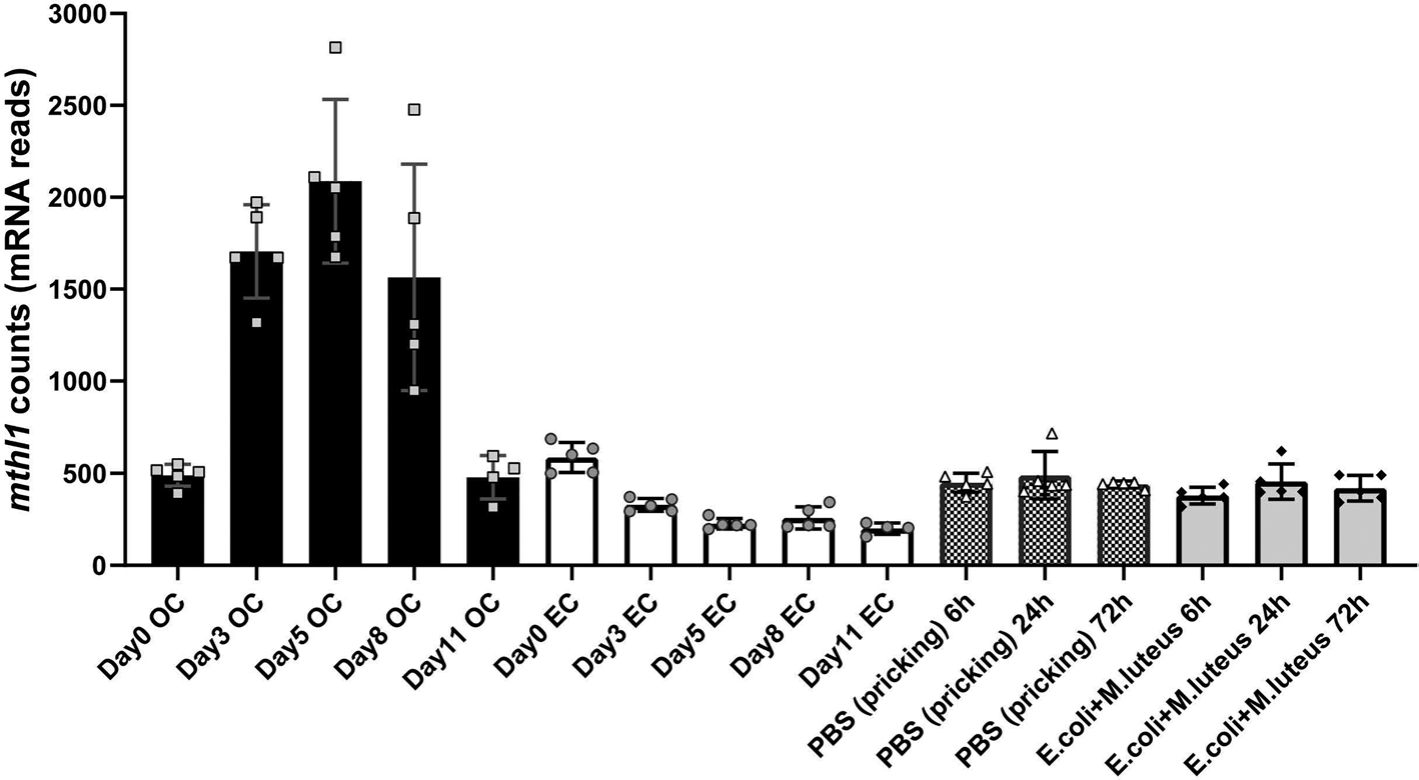
*mthl1* expression level can be induced by oncogenic cell injection but not embryonic cell injection or bacterial infection in *Drosophila*. Wild-type *W^1118^* flies receiving EC/OC injection or buffer PBS/mixture of gram-positive bacteria *Micrococcus luteus* and gram-negative bacteria *Escherichia coli* pricking were collected at the indicated time points and sent for deep sequencing. The average count value of *mthl1* in various groups is presented here. Each dot represents the count value of *mthl1* in one bioreplicate. Each bioreplicate includes 20 flies. Each bar represents an average value from at least four bioreplicates.

As shown in our previous study, the expression of *mthl1* in host flies is induced by injection of oncogenic cells (together with 1,755 other genes) and as apparent from the present data it contributes to the suppression of the proliferation of the injected OCs. We wondered if this induction is relatively specific to the response to injected OCs. We therefore analyzed the expression level of *mthl1* in host flies receiving OC or EC injection or bacterial/virus infections. As documented in Fig. 3, in our deep sequencing data, the count value (representing the relative mRNA expression level) of *mthl1* started to increase sharply in wild-type flies at day 3 p.i., then reached a peak at day 5 p.i. and moderately declined afterward. In contrast, the count value of *mthl1* neither increased in flies receiving EC injection (Fig. 3) nor in flies infected by microbes, namely bacteria (Fig. 3) and viruses (*SI Appendix*, Fig. S2*A*) at various time points. As a reminder, in our previous deep sequencing study, we had dissected whole flies 11 d after OC injection and sorted the OCs based on their GFP marker. The objective then was to better differentiate the transcriptomic profiles of host flies from the injected oncogenic cells themselves. In these experiments, we collected therefore the transcriptomic profiles of the GFP-negative cells as representative for the host flies. We observed (*SI Appendix*, Fig. S2*B*) that the expression level of *mthl1* was relatively low both in the vitro dish–cultured OCs (before injection) and in the OCs sorted from flies 11 d after injection: Clearly, among the sorted cells, the overall signal of *mthl1* is much higher in the host cells than in the injected cells and their descendants. We conclude that the increased expression of *mthl1* from day 3 to day 11 occurs primarily in the host flies rather than in the oncogenic cells.

In parallel, we found that the expression of *mthl1* in the host flies [which affects the proliferation of the injected OCs (cf. Fig. 2)] influences neither the pathogen load nor the survival of flies infected by bacteria (*M. luteus*, *E. coli* and *Enterococcus faecalis*) or viruses (DCV and VSV) (*SI Appendix*, Fig. S3 *A*–*G*).

Collectively, these data suggest that the host’s expression of *mthl1*, which was induced by OCs, is involved in the fly response targeting the proliferation of injected OCs.

### RNA Expression Profiles in *mthl1* LOF Flies after OC Injection and GO Analysis of *mthl1*-Dependent Genes.

Next, we analyzed the global RNA expression profiles in *mthl1* LOF flies on day 3 p.i. For this, we first normalized the expression of all genes in *mthl1* LOF flies after injection of OCs, to that of EC-injected *mthl1* LOF flies. The same approach was applied to the control wild-type *W^1118^* flies. The differentially expressed genes (Log_2_ ≥ 1) in both groups were then compared to determine the genes depending on *mthl1* expression. We thus identified 1216 genes, of which 618 were induced in wild-type flies by injection of OCs but whose induction was blocked in *mthl1* LOF flies. In contrast, the expression of 598 genes was induced by OCs in *mthl1* LOF flies. We then performed GO analysis on genes positively regulated by *mthl1* (the 618 genes) and negatively regulated by *mthl1* (the 598 genes) respectively.

The GO analysis of these genes is somewhat hampered by the fact that close to 50% are still marked in FlyBase only by their gene numbers, which probably reflect the fact that this subfield of physiology is as yet less explored than other domains in flies.

**Fig. 4.**
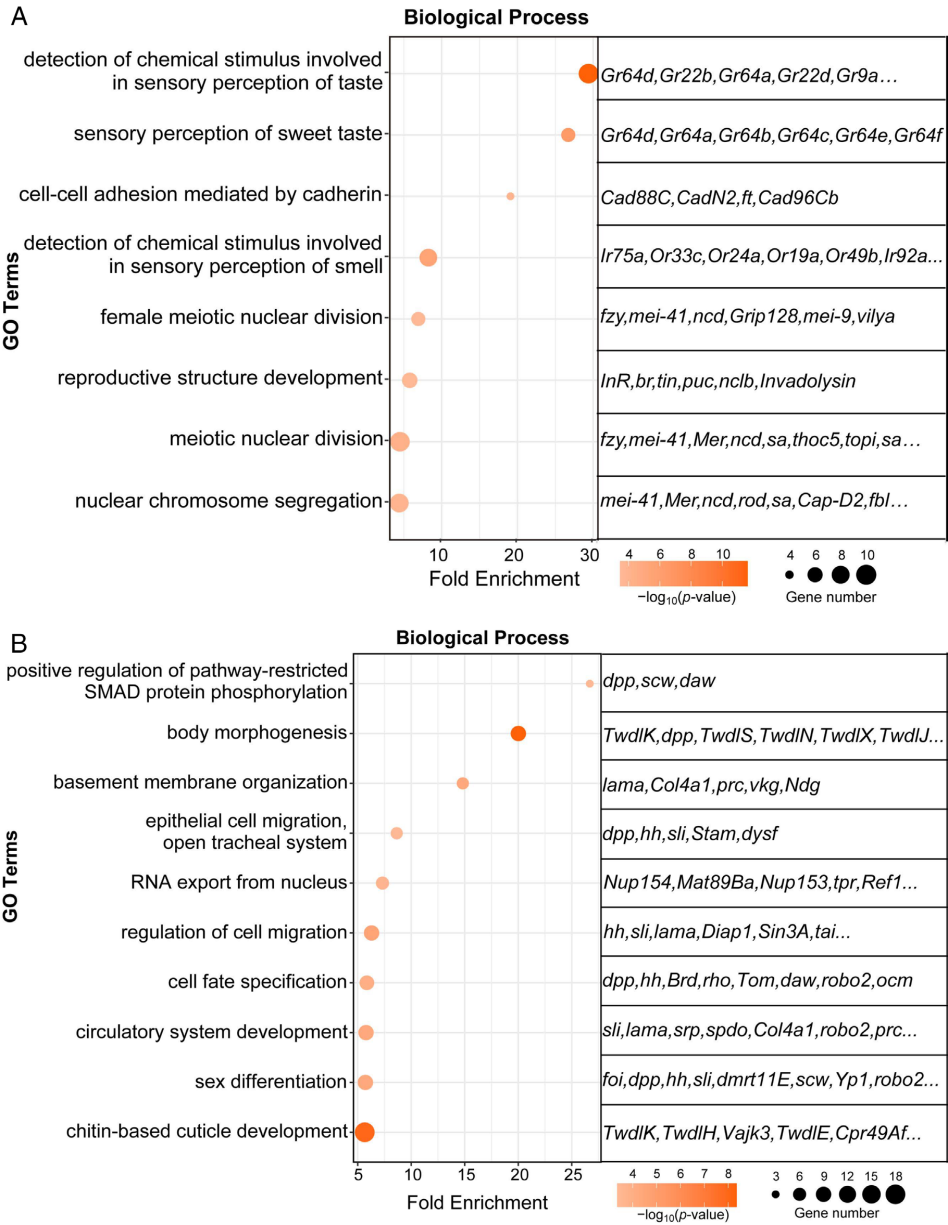
Biological process of GO analysis for *mthl1*-dependent genes. *W^1118^* flies and *mthl1* LOF flies receiving ECs or OCs were collected 3 d after injection for deep sequencing. For each experimental group, four bioreplicates (20 flies/bioreplicate) were collected. Genes positively (*A*) or negatively (*B*) regulated by *mthl1* were analyzed by gene ontology analysis. The ∼10 most enriched BP terms were retrieved after manual curation of the redundancy. For display and clustering, both the −log_10_ (*P*-value) and gene numbers are indicated. Some representative genes corresponding to each GO term are also shown.

According to biological process (BP), prominent among the *mthl1* positively regulated genes were those coding for “sensory perception of taste”, namely chemoreceptors from various families, including Grs and Irs. Most of these genes were also listed in terms of “sweet taste receptor activity,” “ligand-gated ion channel activity,” which are top ranking terms in molecular function (MF) GO analysis (determined by *P* value) (Fig. 4*A* and *SI Appendix*, Fig. S4*A*). Further, the following genes were also positively regulated by *mthl1*: genes of the cadherin family, including *Cadherin 88C* (*Cad88C*), *Cadherin-N2* (*CadN2*), *Cadherin 96Cb* (*Cad96Cb*) as well as *fat* (*ft*), the receptor of Hippo signaling.

Most genes among the *mthl1* negatively regulated group were related to development. This was particularly the case for the genes encoding ligands and regulators of several pathways, such as *decapentaplegic* (*dpp*), *hedgehog* (*hh*), *Slit* (*sli*), *Sin3A* and *Bearded* (*Brd*). We also noted in this category, some genes involved in chitin-based cuticle development, such as members of the Tweedle family (*TwdlC, J, K, N,* etc.) and Cuticular protein family members (e.g., *Cpr49Af*). Other genes also negatively regulated by *mthl1* are known to be involved in morphogenesis and various organ developments, such as *Roundabout 2* (*robo2), lamina ancestor* (*lama*); *fear-of-intimacy* (*foi*); *serpent* (*srp*) etc (Fig. 4*B* and *SI Appendix*, Fig. S4*B*).

### Loss of Function of mthl1 Prolongs the Longevity of *Drosophila*.

**Fig. 5.**
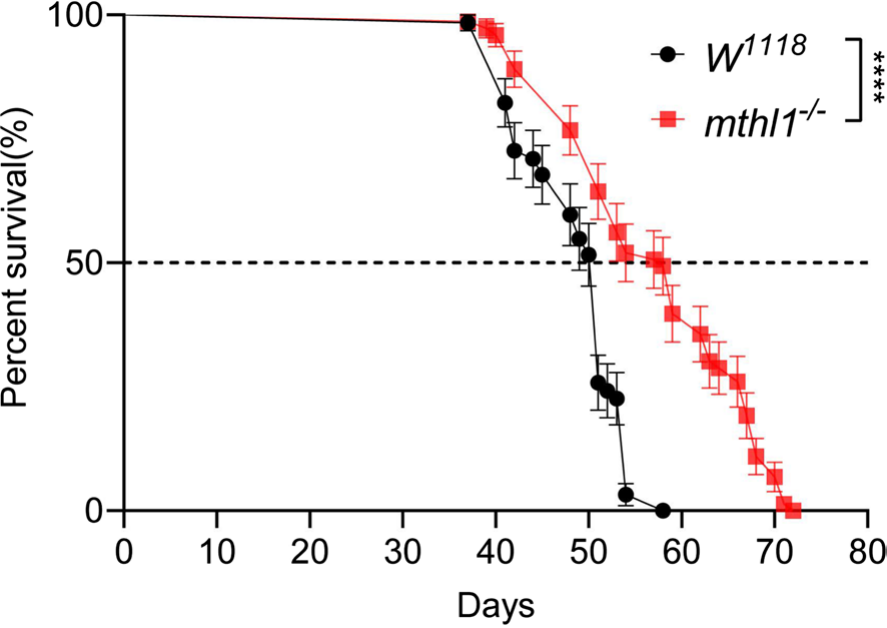
Loss of function of *mthl1* increases the longevity of *Drosophila*. *mthl1* LOF flies and *W^1118^* wild-type flies that hatched in the same day were collected and reared in the same condition with no treatment. Their survival rates were monitored every day until all flies were succumbed. The data represent a sum of three independent experiments, each experiment includes 15 to 20 flies. Long-rank (Mantel Cox) test was used for statistical analysis: ****P < 0.0001.

Two genes from the *methuselah* family, namely *methuselah* (*mth*) and *methuselah like 10* (*mthl10*) were reported to be involved in longevity (16, 17). Here, we observed that in the *mthl1* LOF flies, the longevity of the flies was also significantly prolonged (Fig. 5) with a LT50 increase ~8 d and an end point of ~14 d. This extends the initial report by Benzer and the other group of the other members of the methuselah gene family (16–20).

### The Mammalian Genome Contains Several Potential Homologues of *mthl1* Which Can Be Induced in Mice Tissues by Injection of Melanoma Cells.

**Fig. 6.**
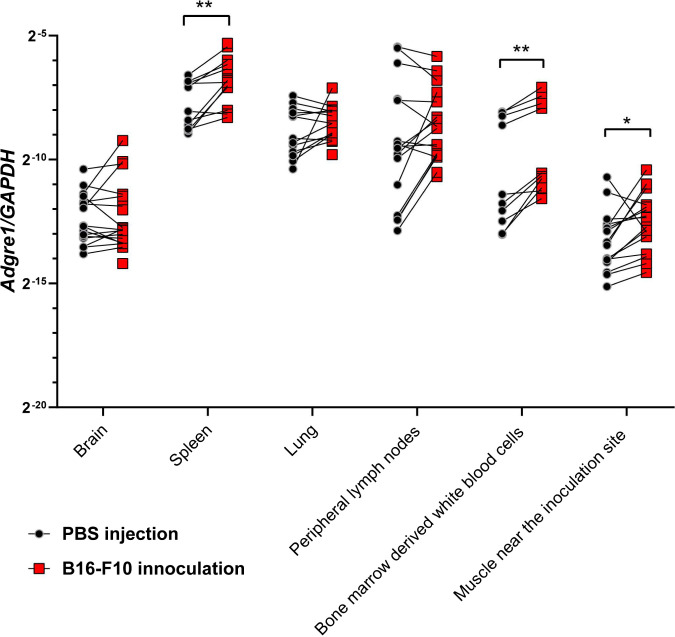
The expression of *Adgre1* in various tissues/organs of mice was up-regulated in response to injected B16-F10 melanoma cells. 2 × 10^5^ B16-F10 cells were subcutaneously inoculated into C57/BL6 mice. Three days later, mice were killed and the total RNA of indicated tissues/organs was collected for RT-qPCR. The relative expression level of *Adgre1* on tumor-bearing mice relative to buffer (PBS)-injected mice was obtained after technical normalization with their internal control (*GAPDH*). Each dot represents the *Adgre1* relative expression in the indicated tissue/organ from one mouse. The data were collected from five independent experiments. Each experiment includes at least 3 mice in each group. Each experimental group includes 12 to 18 mice in total. Paired two-tailed Student’s *t* test was used for statistical analysis: *P < 0.05; **P < 0.01.

Based on the sequence similarities, seven adhesion G-protein-coupled receptors in mice appeared as potential mammalian homologues of *mthl1*. Those are namely *Adgre1* (highest sequence similarity) and to a lesser extent *Adgre5*, *Adgrf2*, *Adgrf3*, *Adgrf4*, *Adgrd1,* and *Adgrd2* (cf. FlyBase and NCBI). We were curious if these potential mammalian homologues of *mthl1* can be induced by mammalian tumor cells in mice tissues, just like *mthl1* in *Drosophila*. To address this question, we choose the C57/BL6 mouse model for intradermal inoculation (inner side of the right thigh) of B16-F10 melanoma cells (a highly malignant cancer cell line with a constantly overactivated Ras pathway) (21, 22). Three days after inoculation, before the melanoma popped up at the inoculation site, the mice were killed, and various organs were collected for detection of the mRNA expression levels of the potential mammalian homologues of *mthl1.* As demonstrated in Fig. 6, *Adgre1,* which is the potential mammalian homologue of *mthl1* with the highest sequence similarity, was significantly up-regulated upon melanoma cell injections (as compared to control), in samples from spleen and from white blood cells derived from bone marrow. The same tendency was also found in other samples although not statistically significant. The same holds true for other genes like *Adgre5*, *Adgrf2, Adgrf3*, *Adgrf4,* and *Adgrd1*, which were slightly up-regulated, but the variations were too large to be conclusive (*SI Appendix*, Fig. S5).

## Discussion

The results presented here call for several comments, in particular regarding the following points:

(1) In our previous study, we noticed the proliferation of injected OCs is very limited during day 0 to day 4 p.i., (referred to as lag period here), yet there is a strong response in terms of transcriptomic activity at day 3 p.i.

In this study, we observed that in *mthl1* LOF flies, the injection resulted in a rapid and significant increase in proliferation of injected OCs in the flies. In keeping with this result, overexpression of *mthl1* suppressed significantly the proliferation of the injected OCs. These results indicate that MTHL1 is a major regulator of the proliferation of OCs and point to a clear role of this receptor in a targeted anti-cancer defense reaction. At this stage, we do not exclude that other factors contribute to this antiproliferative role of *mthl1*.

(2) At the later time point, this *mthl1*-dependent antiproliferative effect is partially overcome, most likely by an important change in the transcriptomic profile of injected OCs as documented in one of our previous studies (Journal of Innate Immunity, 2023) reporting the transcriptomic profiles of injected OCs had evolved for 11 d in the experimental flies following their injection on day 0. We are aware that additional data are required to establish this hypothesis more firmly, which is one of our present priorities.

(3) Whereas OC injection clearly induces *mthl1* expression in the *Drosophila* host, which in turn represses the proliferation of the injected OCs, the same effects are not observed when primary *Drosophila* ECs or pathogenic microbes (namely viruses and gram-positive and -negative bacteria) are injected into flies. This indicates that the ligand(s) of *mthl1* come(s) either directly from the injected OCs or from so far unspecified modifications resulting possibly from distinct immunopathological effects induced by the injection of the OCs.

(4) The transcriptomic analysis of *mthl1*-dependent genes interestingly reveals that many (but not all) chemoreceptors induced by injected OCs in wild-type flies are positively regulated by *mthl1*, point to a chemoreceptors cascade. Their functions need to be further explored, which will of course imply identifications of their respective ligands, another of our present targets.

(5) In parallel, *mthl1* represses the expressions of genes from several important developmental pathways, namely *dpp*, *hh*, *wingless* (*wg*), *Notch* (*N*), etc. As a reminder, the OCs originate from embryonic *Drosophila* cells, in which these developmental pathways are active and crucial for the growth and proliferation of these cells, as discussed in (Chen et al, 2023, ahead of print). Repression of these genes in the injected OCs by the host is thus in keeping with the antiproliferative effect, which we observed during the lag period.

(6) There are seven members from adhesion GPCRs family in mammals predicted to be the homologues of *mthl1* (according to the sequence similarities as noted in FlyBase/NCBI). Among them, *Adgre1* has the highest similarity with *mthl1*. *Adgre1* encodes for F4/80 antigen, which is a widely used marker for monocyte macrophage identified by Austyn and Gordon (23). It is also found to be expressed in some myeloid-derived cells in mice (*eosinophils*, monocytes, macrophages, and dendritic cells) and other mammals, namely pig, human, etc. (24). It is reported to be involved in myeloid-derived immune cells’ development and defense reactions (24, 25). However, the roles of mammalian adhesion GPCRs in response to tumor cells have been poorly investigated. Here, we found that the expression of *Adgre1* in both bone marrow and spleen (nests of immature and mature myeloid-derived immune cells, respectively) is significantly induced by melanoma cell inoculation 3 days p.i. This suggests that one or several adhesion GPCRs may be involved in the early response to injected tumor cells in mice. This raises the exciting hypothesis that innate immune defenses against cancerous cells in flies and mammals share some of their characteristics. More specifically, the injection of tumor cells might increase the population of myeloid-derived macrophages in mice. If validated by future studies, this hypothesis would extend the observations of stringent parallelisms between innate defenses against microbes in flies and mammals documented in earlier studies in the field.

## Materials and Methods

### Fly Strains.

Flies were grown on standard cornmeal-yeast-agar medium at 25 °C with 60% humidity. All fly lines used in the study were Wolbachia, Nora, DCV, VSV and Microsporidia free. *W^1118^* flies were used as wild-type control, *mthl1* loss-of-function flies (BL18976) and *UAS-LacZ* (BL8529) flies were obtained from Bloomington Drosophila Stock Center (BDSC), for *UAS-mthl1,* cDNA encoding *mthl1* were cloned into the *pUAST-attB* vector in Sino-French Hoffmann Institute.

### Cell Culture.

The RasV12-GFP oncogenic cells were grown as precious descripted (15). B16-F10 cells were grown in RPMI-1640 medium (Sigma-Aldrich) plus nonessential amino acids (Sigma-Aldrich), 10% FBS (Gibco), 1% penicillin/streptomycin (Invitrogen) in 37 °C, 5% CO_2_.

Methods for injection of RasV12 GFP oncogenic cells into flies was descripted previously (15).

### 10X Visium Spatial Transcriptomics Materials.

*W^1118^* flies were collected and snap-frozen in an optimum cutting temperature (OCT) compound (SAKURA, Cat#: 4583) chilled by liquid nitrogen. The frozen flies were cut in a precooled cryostat with 10-μm thickness and systematically placed on chilled Visium Tissue Optimization Slides (3000394, 10× Genomics) for capture area selection. Once the capture area is determined, one more section was taken from the frozen flies and was placed on the Visium Spatial Gene Expression Slides (2000233, 10× Genomics) with a 6.5 × 6.5-mm capture area, which contains 5,000 clusters (spots) with barcoded primers (10× Genomics). The primers are attached to the slide by the 5′ end and contain a cleavage site, a T7 promoter region, a partial read1 Illumina handle, a spot-unique spatial barcode, a unique molecular identifier, and a sequence of 20 thymine linked with one adenine or cytosine or guanine and one random DNA. Reverse transcription (RT) was conducted as previously described (26) to synthesize barcoded cDNA. After RT, wells were washed with 0.1× saline-sodium citrate buffer once, then incubated at 56 °C with interval shaking for 1.5 h in a tissue removal mix of proteinase K (QIAGEN) and PKD buffer (QIAGEN, pH 7.5) at a ratio of 1:1. The spatially barcoded cDNA was enzymatically released as previously described (26) and were collected and transferred to 96-well plates for spatial transcriptomic library preparation with an automated MBS 8000 system (27) for second-strand cDNA synthesis. The second-strand cDNA was then used for the in vitro transcription, adaptor ligation, and a second RT to construct the library. Sequencing handles and indexes were added in an indexing PCR reaction, and the finished libraries were purified and quantified as previously described (28). Sequencing was performed on the Illumina NovaSeq 6000 with a sequencing depth of at least 50,000 reads per spot and 150-bp paired-end (PE150) reads (performed by Biomarker Technologies Corporation, Beijing, China).

### RNA Analysis.

Total RNAs from whole fly and tissues of mice or flies were isolated using RNAex pro Reagent (AG) and analyzed as previously described (15). The sequences of primers are shown in *SI Appendix*, Table S1. The RNA deep sequencing was delivered in Ribobio Co. Ltd (Ribobio, China).

### Inoculation of Melanoma B16-F10 Cells into Mice.

C57/BL6 mice were fed with standard rodent chow diet and water ad libitum and were given 1 week to acclimatize after their delivery to the animal facility. The animals were maintained at 22 to 24 °C with 12-h light cycle in a specific pathogen-free environment. 2 × 10^5^ B16-F10 cells were subcutaneously inoculated into the inner right hind leg of each mouse. PBS was injected into the same spot of each mouse as control. Four days later, we killed and dissected these mice and collected indicated tissues/organs/cells for total RNA extraction and RT-PCR. This animal experiment was delivered in GuangZhou ExoDiag company.

### Statistical Analysis.

Student’s *t* test was used for statistical analysis of data with GraphPad Prism (GraphPad Software). Error bars indicate SEM. Survival curves were plotted and analyzed by log-rank analysis (Kaplan–Meier method) using GraphPad Prism (GraphPad Software). *P*-values lower than 0.05 were considered statistically significant.

### Isolation of D11 Cells, Host Cells, and Embryonic Cells.

After injection of oncogenic cells, the flies were incubated at 25 °C for 11 d to let the injected cells grow inside the fly body. While the first isolated cell suspension was kept on ice, the remaining tissues were further homogenized in the dissociation buffer with 0.5 mg/mL liberase enzyme (Sigma) at room temperature for 15 min. The dissociated tissues were repeatedly washed by PBS, and the remaining cells were harvested. All collected cells were stained with DAPI to discriminate dead cells and applied on a BD FACS Arria II cell sorter. The sorted GFP+DAPI cells (labeled as in vivo D11 OC) and GFP-DAPI cells (labeled as Host) were immediately dissolved in TRIzol (Invitrogen) for the subsequent total RNA isolation and RNAseq analysis. Embryos from at least 500 transgenic flies (Bloomington: BL1691) were collected, bleached (3 to 5 min to remove chorion membranes) and washed with phosphate-buffered saline (PBS), then placed within a cell strainer (40 μm, Falcon) and homogenized within the PBS solution using the white end of the plunger (sometimes inside the protective cap) of an insulin injector. The embryonic cell suspension was then purified using Ficoll Paque Plus solution (GE Healthcare). After centrifugation at 400 g for 30 min, the alive cells located in the interphase were collected and resuspended in PBS.

### Gene Ontology Enrichment Analysis.

Genes positively regulated and negatively regulated by *mthl1* were used to perform GO analysis. The GO analysis covers three items: cellular component, MF, and BP (http://www.geneontology.org/).

## Supplementary Material

Appendix 01 (PDF)

## Data Availability

The RNA sequencing data of *W^1118^* flies have been deposited at NCBI SRA at the reference number of PRJNA686503 (15). The RNA sequencing data of *mthl1* LOF flies and the spatial transcriptomic RNA-seq data reported in this paper have been deposited in the Genome Sequence Archive in National Genomics Data Center. China National Center for Bioinformation / Beijing Institute of Genomics. Chinese Academy of Sciences (GSA: CRA010023) that are publicly accessible at https://ngdc.cncb.ac.cn/gsa (29).
