# Supplementary material for: mthl1, a potential Drosophila homologue of mammalian adhesion GPCRs, is involved in antitumor reactions to injected oncogenic cells in flies
Source: Proc Natl Acad Sci U S A. 2023 Jul 17;120(30):e2303462120. doi: 10.1073/pnas.2303462120 (PMC10374174; doi:10.1073/pnas.2303462120)
Supplement: Supplementary file 1 — Appendix 01 (PDF) [file pnas.2303462120.sapp.pdf]

Supplementary Information for

mthl1, a Drosophila homologue of Mammalian adhesion GPCRs, is involved in antitumor reactions to injected oncogenic cells in flies.

Di Chen [a, *†], Xiao Lan [a†], Xiaoming Huang [a], Jieqing Huang [a], Xiaojing Zhou [a], Jiyong Liu [a], Jules A Hoffmann[* a, b, c]

[a] Sino-French Hoffmann Institute, School of Basic Medical Science, Guangzhou Medical University, Guangzhou, 511436, China.

[b] University of Strasbourg Institute for Advanced Study;

[c] Institute of Molecular and Cellular Biology (IBMC), CNRS, Insect Models of Innate Immunity (M3I; UPR9022), F-67084 Strasbourg, France.

†Co-first authors: Di Chen, Xiao Lan

*Corresponding Authors:

1 Jules A. HOFFMANN, University of Strasbourg Institute for Advanced Study;

Institute of Molecular and Cellular Biology (IBMC), CNRS, Insect Models of Innate Immunity (M3I; UPR9022), F-67084 Strasbourg, France.

Tel: +00 33 6 25 02 11 14

E-mail: j.hoffmann@unistra.fr

2 Di CHEN, Sino-French Hoffmann Institute, School of Basic Medical Science, Guangzhou Medical University, Guangzhou, 511436, China.

Tel: +86 13533202789

E-mail: chenseptember@outlook.com

This PDF file includes:

Supplementary text
Figures S1 to S5
Tables S1 to S3

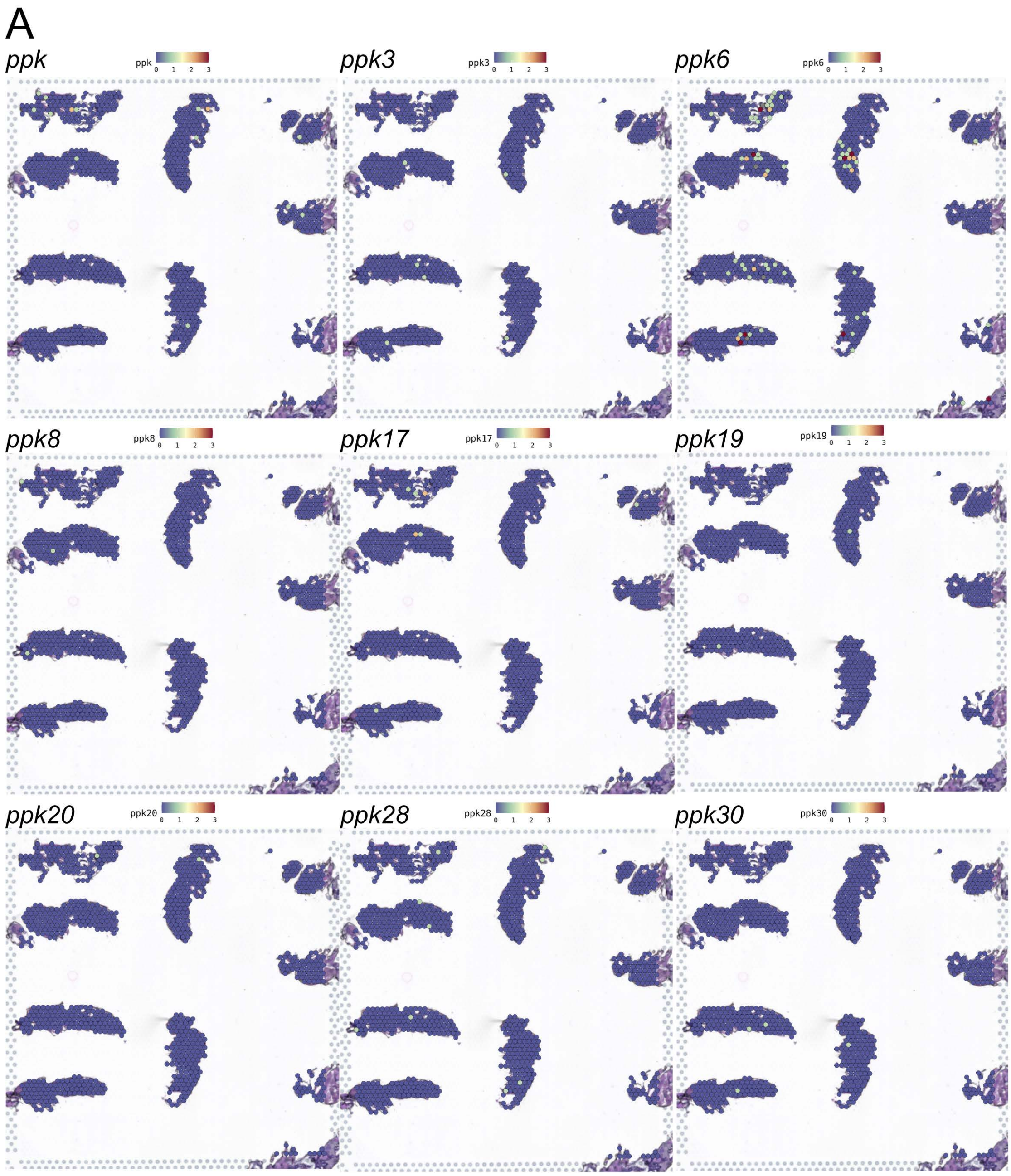
A
ppk
ppk 0 1 2 3
ppk3
ppk3 0 1 2 3
ppk6
ppk6 0 1 2 3
ppk8
ppk8 0 1 2 3
ppk17
ppk17 0 1 2 3
ppk19
ppk19 0 1 2 3
ppk20
ppk20 0 1 2 3
ppk28
ppk28 0 1 2 3
ppk30
ppk30 0 1 2 3

B

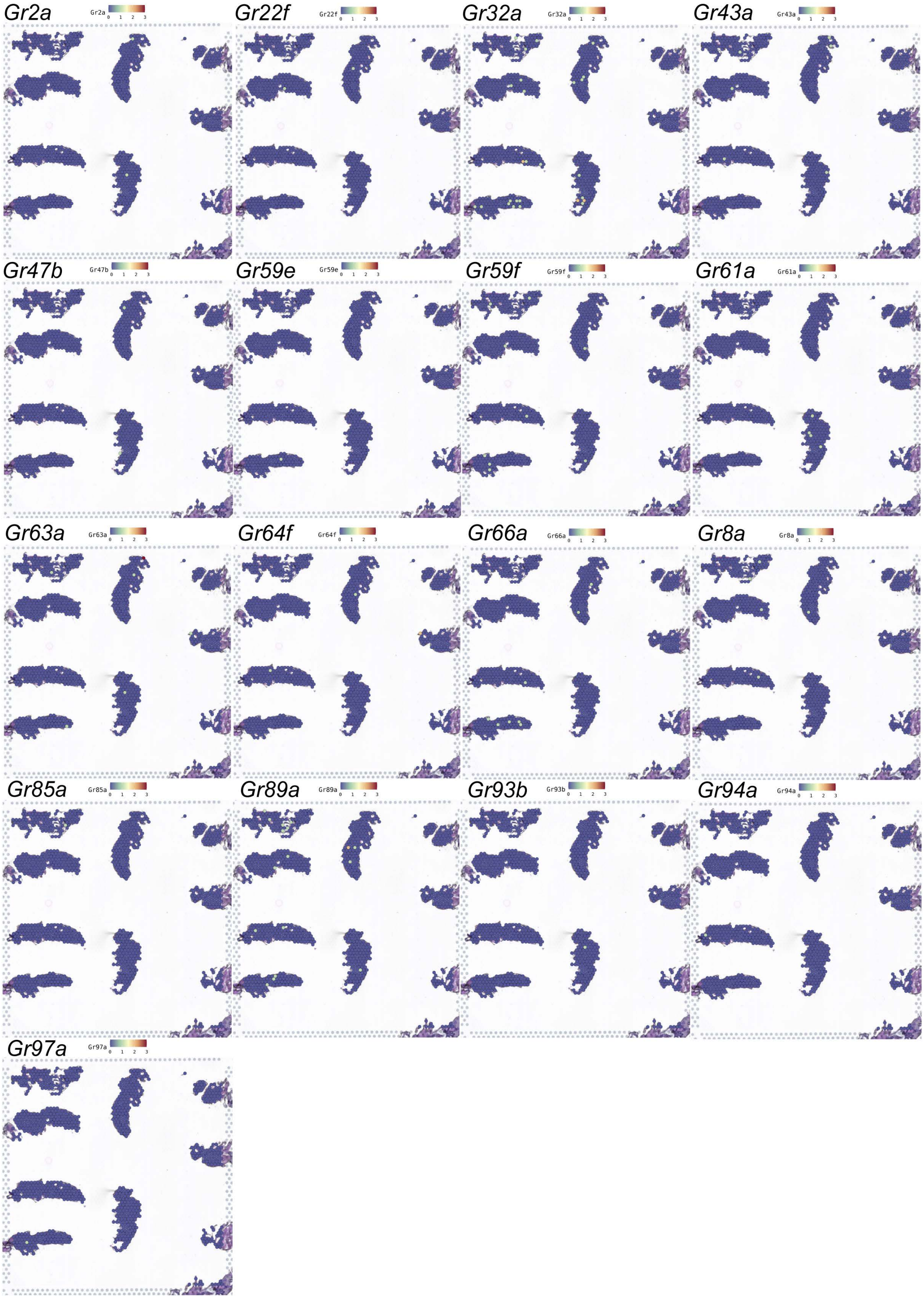
Gr2a
Gr22f
Gr32a
Gr43a
Gr47b
Gr59e
Gr59f
Gr61a
Gr63a
Gr64f
Gr66a
Gr8a
Gr85a
Gr89a
Gr93b
Gr94a
Gr97a

C

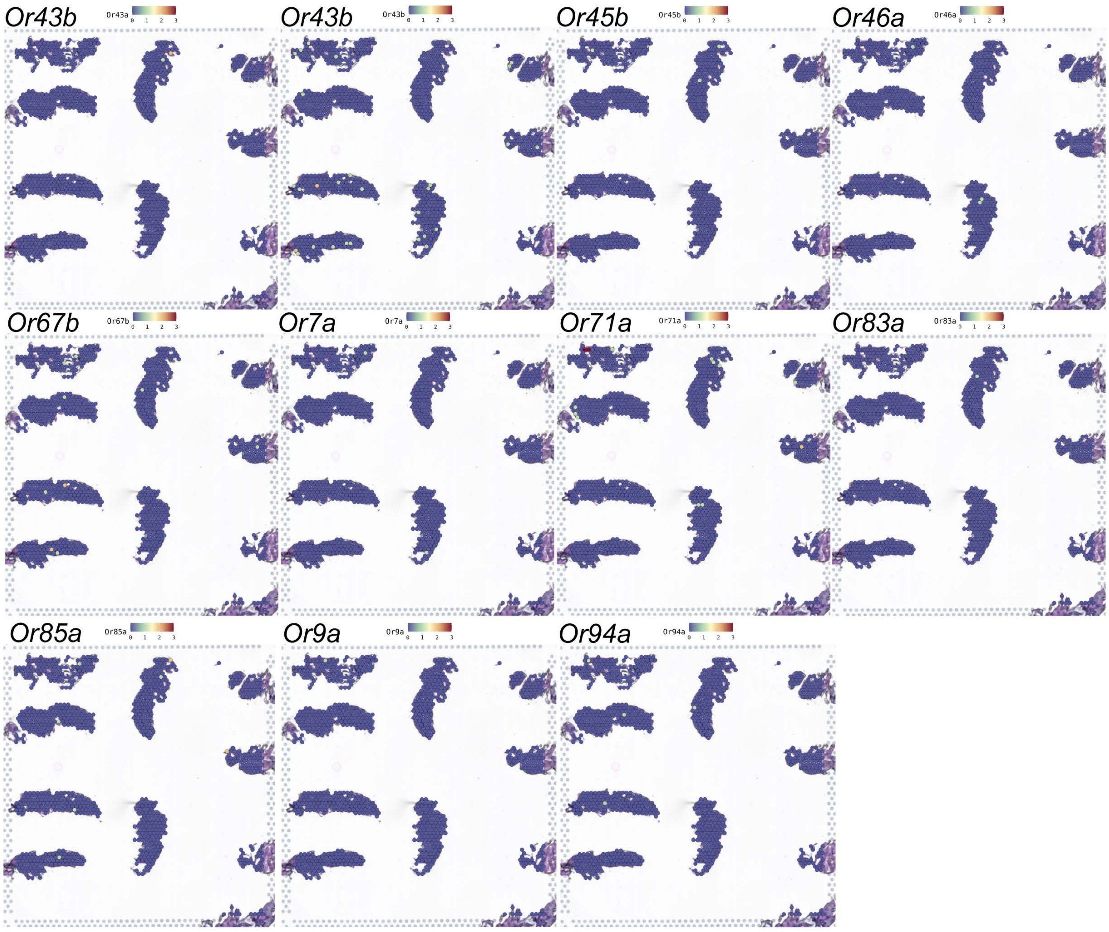

Or43b
Or43b
Or45b
Or46a
Or67b
Or7a
Or71a
Or83a
Or85a
Or9a
Or94a

D

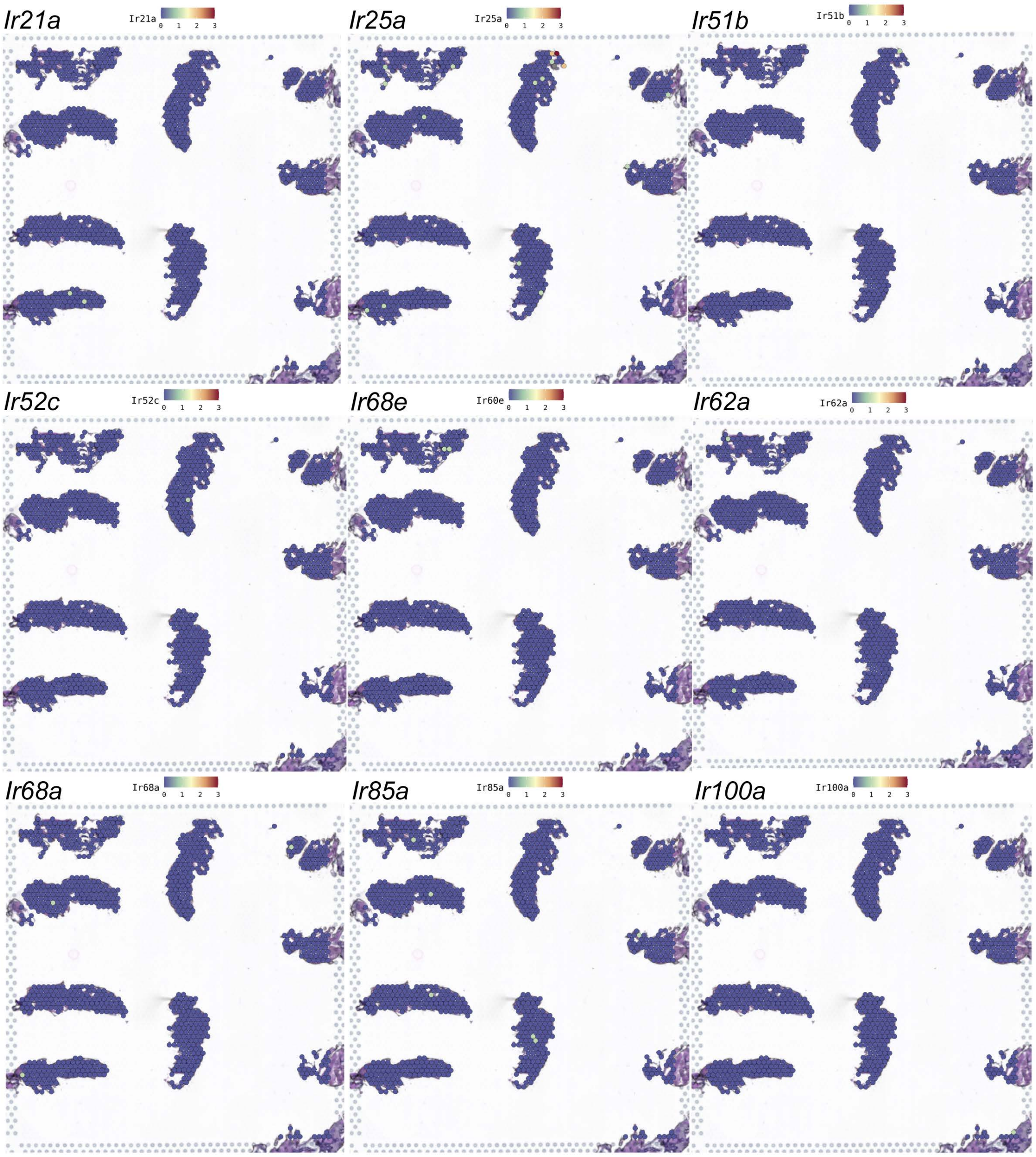

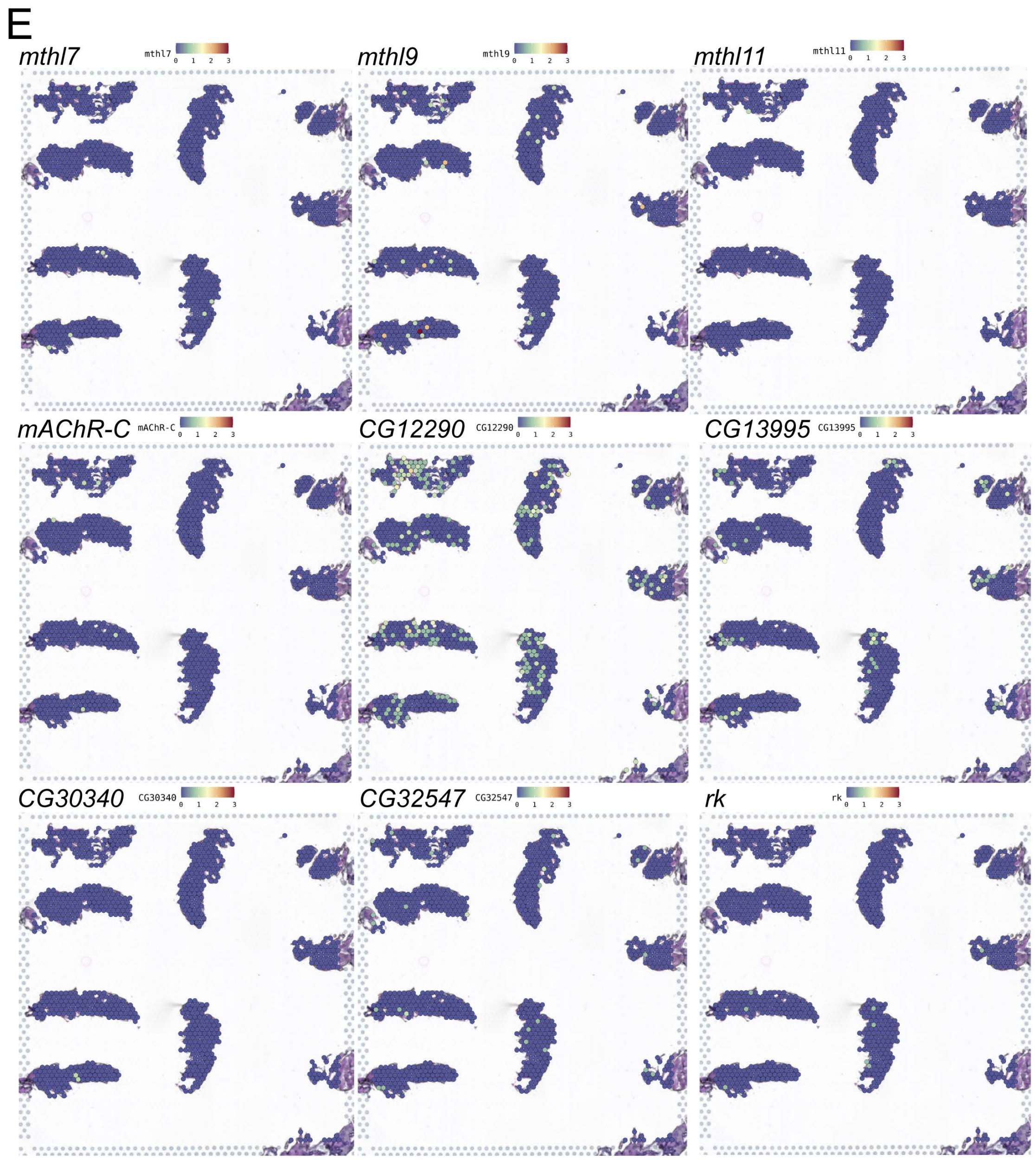

Supplementary Figure 1. Feature plots analysis for chemoreceptors induced by injected OCs from 5 different families.

10 male W^{1118} wild type flies receiving OCs injection 3 days p.i. were randomly collected and fixed in the embedding box with optimal cutting temperature compound (OCT), then cryo-sectioned in a thickness of 10um/slide for the spatial transcriptomic sequencing. The distributions of indicated chemoreceptors were visualized using characteristic feature plots analysis. The relative expression level of different chemoreceptors is indicated by the color of

the scale bar (the lowest expression level is set as value 0 in blue and the highest expression level is set as 3 in red).

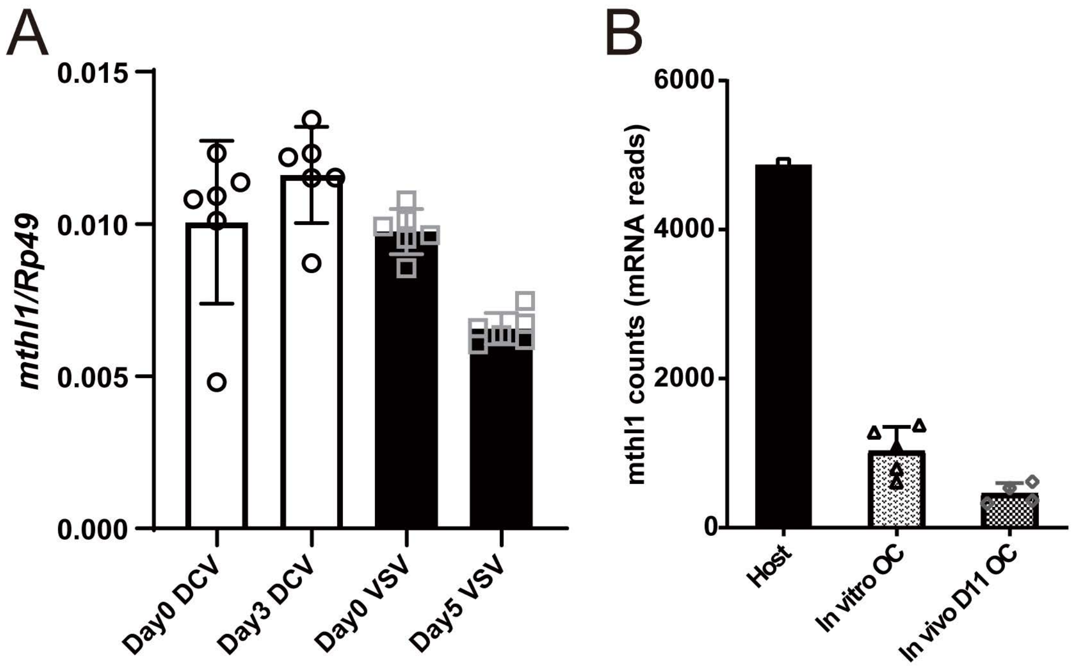

Supplementary figure 2. The expression of *mthl1* is not induced in OCs or in host *Drosophila* infected by virus.

(A)*mthl1* expression level in *mthl1* LOF flies and W^{1118} wild type flies infected by *Drosophila* C Virus (DCV) or Vesicular Stomatitis Virus (VSV). The total RNAs of flies in various experimental groups were collected at the indicated time point after infection for RT-PCR. The relative expression level of *mthl1* after normalization to house-keeping gene *Ribosomal protein 49* (*Rp49*) is presented here. Each dot represents the relative expression level of *mthl1* from one bio-replicate. Each bar represents an average value from at least 4 bio-replicates. The data were collected from two independent experiments.

(B) Dish culture OCs, cells from host *Drosophila* (GFP^-) and cells sorted from *Drosophila* 11 days after injection (GFP^+) were collected and sent for deep sequencing, The averaged count value of *mthl1* in various groups was collected and presented here. Each dot represents the count value of *mthl1* from one bio-replicate. Each bar represents an average value from at least 4 bio-replicates. Each bio-replicate includes 20 flies. The count value of cells from host *Drosophila* 11 days after injection is collected from one bio-replicate that includes 100 flies.

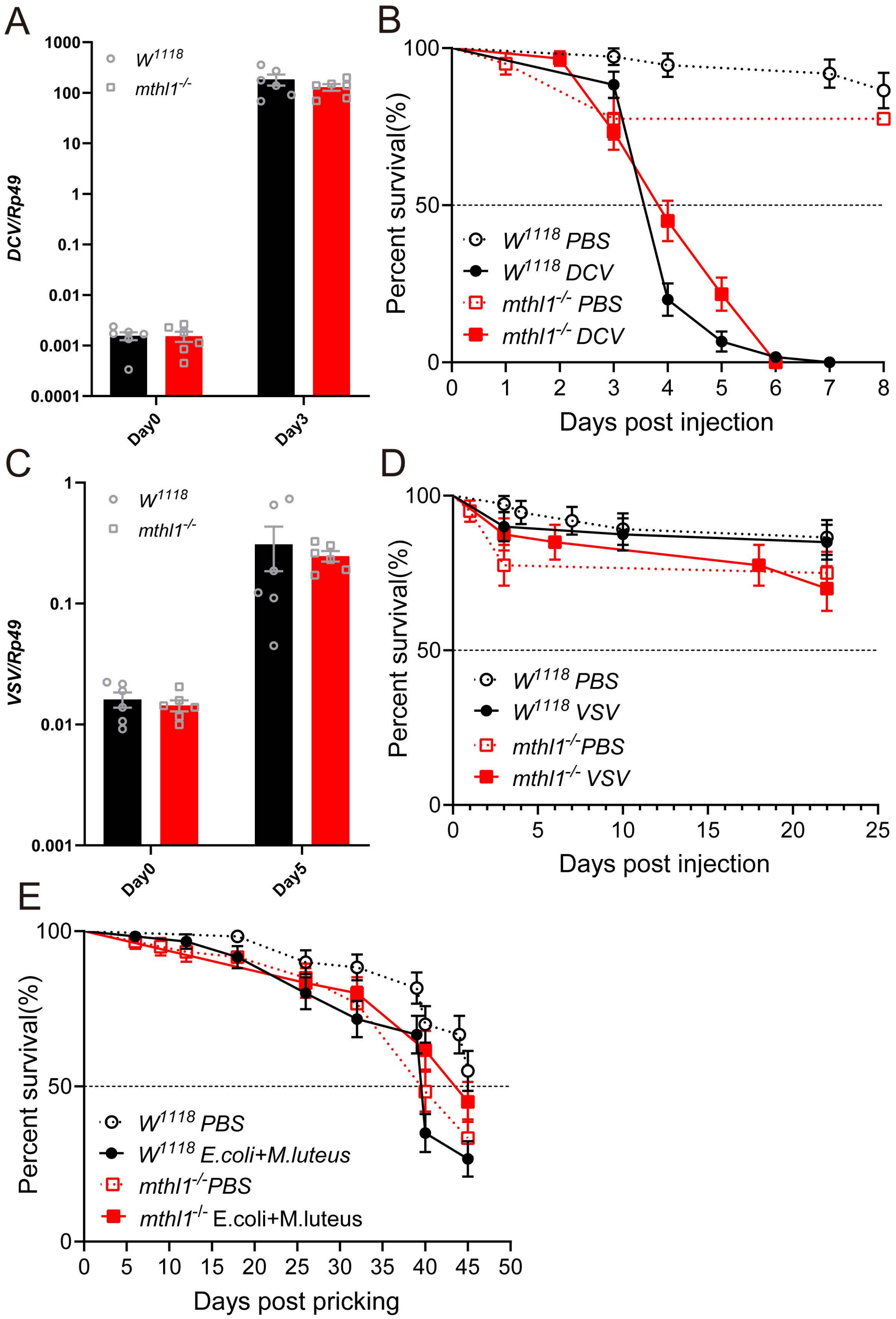
A
1000
100
10
1
0.1
0.01
0.001
0.0001
DCV/Rp49
W1118
mthl1-/-
Day0
Day3
B
Percent survival(%)
100
50
0
W1118 PBS
W1118 DCV
mthl1-/- PBS
mthl1-/- DCV
0 1 2 3 4 5 6 7 8
Days post injection
C
1
0.1
0.01
0.001
VSV/Rp49
W1118
mthl1-/-
Day0
Day5
D
Percent survival(%)
100
50
0
W1118 PBS
W1118 VSV
mthl1-/-PBS
mthl1-/- VSV
0 5 10 15 20 25
Days post injection
E
Percent survival(%)
100
50
0
W1118 PBS
W1118 E.coli+M.luteus
mthl1-/-PBS
mthl1-/- E.coli+M.luteus
0 5 10 15 20 25 30 35 40 45 50
Days post pricking

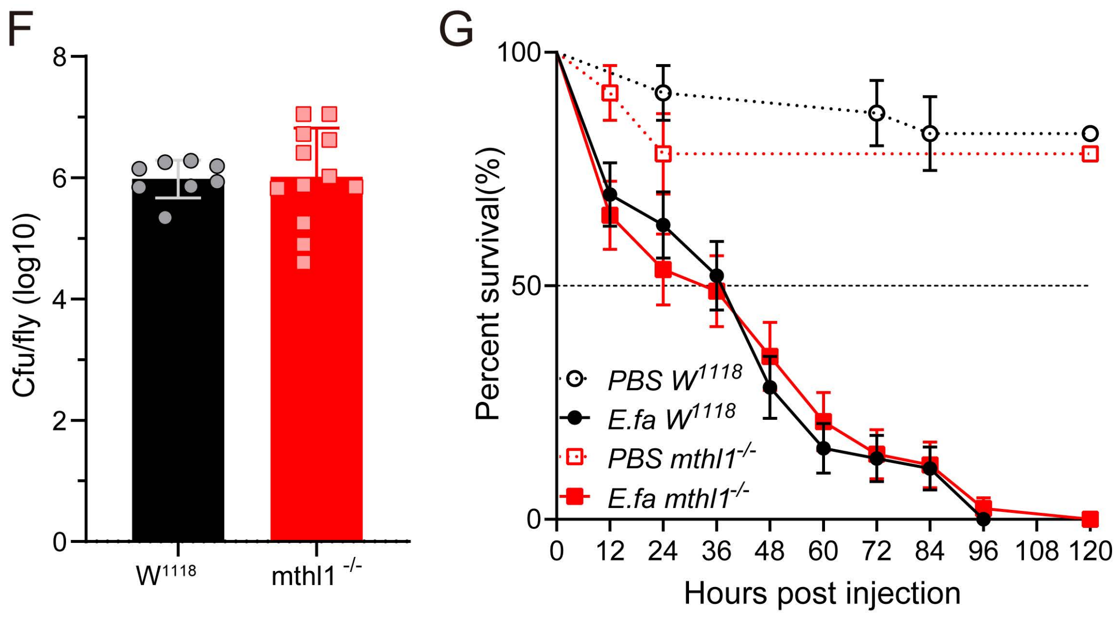

Supplementary figure 3. *mthl1* does not affect the pathogen load and host survival upon bacterial and viral infection in *Drosophila*.

(A) DCV load and (B) survival rate of W^{1118} wild type flies and *mthl1* LOF flies receiving DCV injection. (C) VSV load and (D) survival rate of W^{1118} wild type flies and *mthl1* LOF flies receiving VSV injection. One data point represents a pool of 6-8 flies. The data were collected from two independent experiments. Each experiment includes 3 bio-replicates. For the survival data, 60 flies in each group were collected after injection of DCV and VSV. The figure represents a sum of two independent experiments. (E) Survival rate of W^{1118} wild type flies and *mthl1* LOF flies pricking by a mixture of Gram-positive bacteria *Micrococcus luteus* (*M. Luteus*) and Gram-negative bacteria *Escherichia coli* (*E. coli*). (F) Bacterial load and (G) survival rate of W^{1118} wild type flies and *mthl1* LOF flies infected by *Enterococcus faecalis* (*E. fa*). One data point represents a pool of 5 flies for the bacteria load. The data were collected from two independent experiments. Each experiment includes 4-6 bio-replicates. For the survival, 45-50 flies were collected from the two experimental groups of two independent experiments.

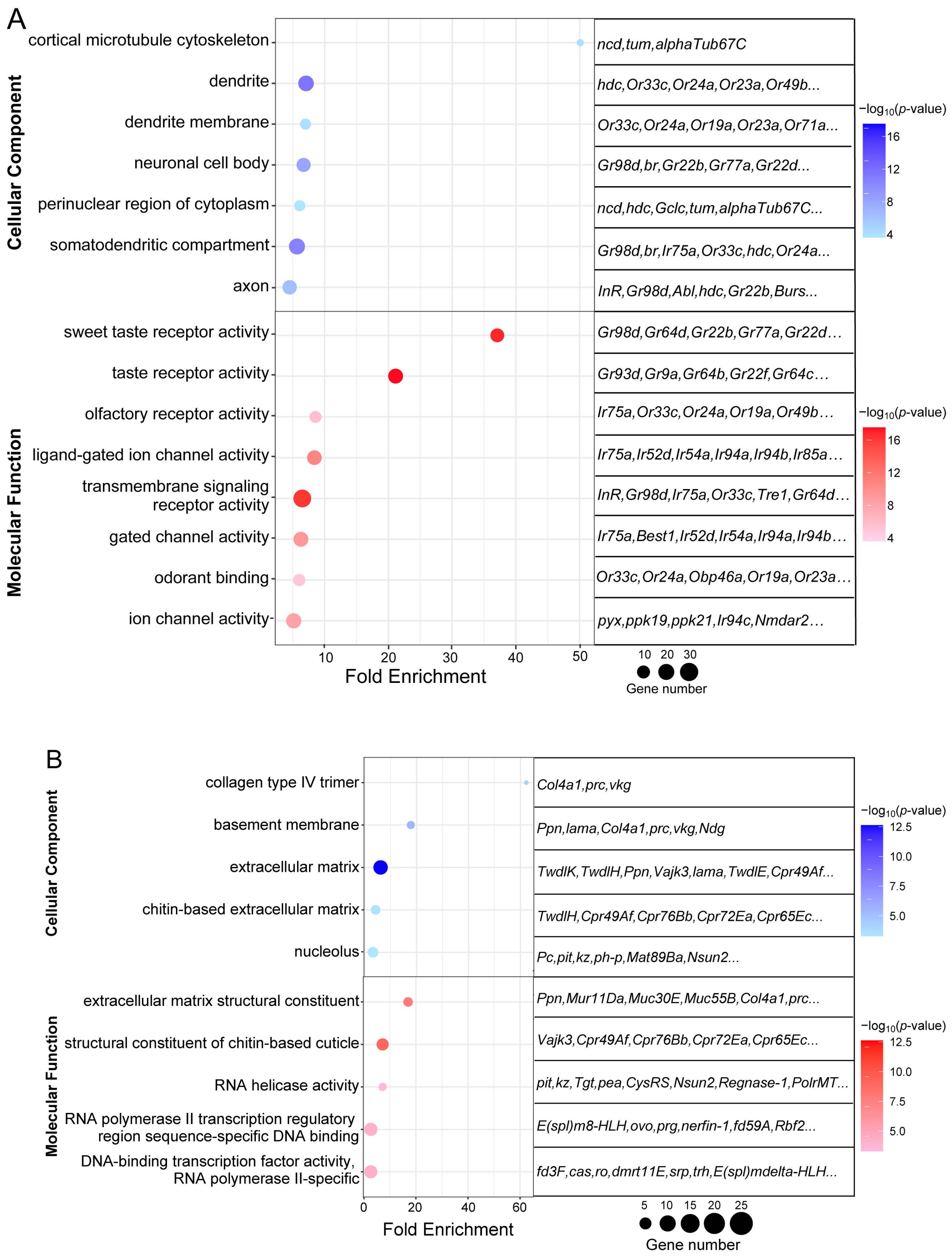

Supplementary figure 4. Cellular Component (CC) and Molecule Function (MF) of GO analysis for *mthl1* regulated genes.

W[1118] flies and *mthl1* LOF flies receiving ECs or OCs were collected 3 days after injection for deep sequencing. For each experimental group, 4 bio-replicates (20 flies/bio-replicate) were collected. Genes positively (A) or negatively (B) regulated by *mthl1* were analyzed by Gene

Ontology analysis. The ~10 most fold enrichment Cellular Component (CC) and Molecule Function (MF) terms were retrieved after manual curation of the redundancy. For display and clustering, both the −log10 (P value) and gene numbers are indicated. Some representative genes corresponding to each GO term are also shown.

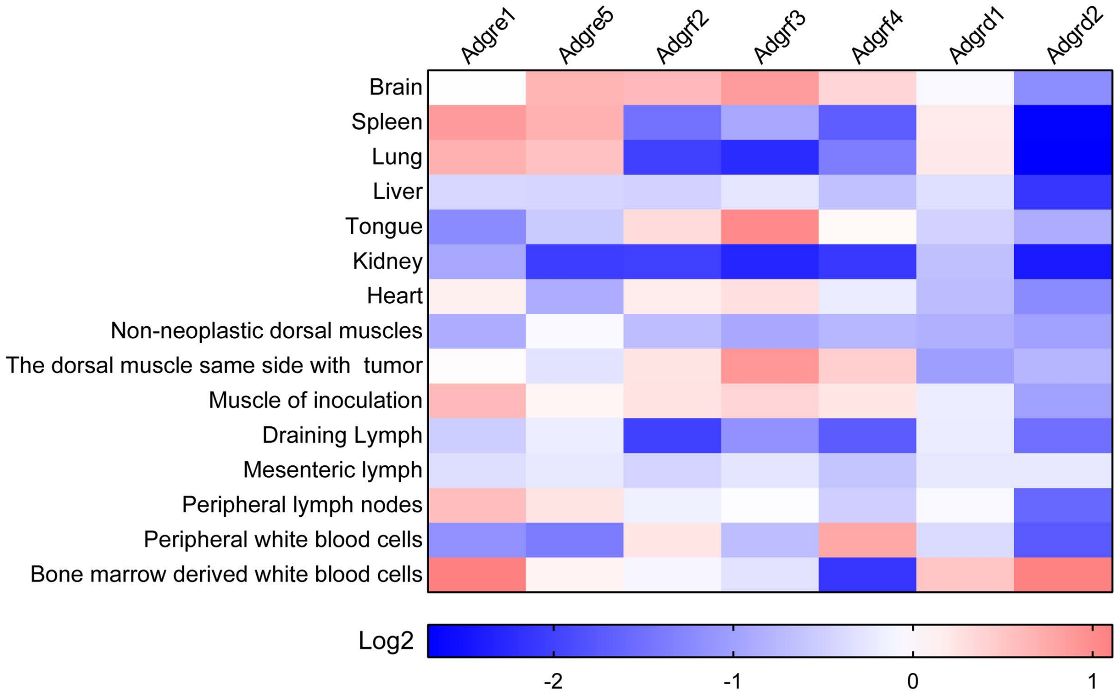

Supplementary Figure 5. The expression of genes homologue to *mthl1* in various organs/tissues of mice upon B16-F10 cells inoculation.

$2X10^5$ B16-F10 cells were epidemically inoculated into C57/BL6 mice. 3 days later, mice were sacrificed and the total RNA of indicated tissues/organs was collected for RT-qPCR. The expression level of indicated genes on tumor bearing mice relative to buffer (PBS) injected mice was obtained after technical normalization with their internal control (*GAPDH*) to determine the fold induction value. The values were log2-transformed and presented by heatmap. The data were collected from 5 independent experiments and each experiment includes at least 3 mice in each group. Each experimental group includes 12-18 mice in total.

Supplementary Table 1
Primer sequences for RT-qPCR

Gene	Forward primer, 5'- sequence -3'	Reverse primer, 5'- sequence -3'
Rp49	GCCGCTTCAAGGGACAGTATCT	AAACGCGGTTCTGCATGAG
GFP	TGTCCTTTTACCAGACAACC	GCAGCTGTTACAAACTCAAG
mthl1	GCCTGAAGCTAGTCGTCGTT	AGCCCACCACGATGAAGATG
GAPDH	GGCCTCCAAGGAGTAAGAAA	GCCCCTCCTGTTATTATGG
Adgre1	TTCAGCTCTCGCAACATCAA	CCCGTCTCTGTATTCAACCA
Adgre5	CTCTTCCTGGGCTCAATCAT	CAGCGTTGCCATGTACTCA
Adgrf2	TGGCTTAATTGGGACATGAC	GGCTCTTACTTCCTGGAACA
Adgrf3	AGGGAGGTTGGTCAGATGAA	AGTGTGTTGGGACATGAGGAT
Adgrf4	CCGGGTGGTTGTTACAGAGA	TGAACGTTGGCACTGAAGTT
Adgrd1	CCTTCAAGCACAAGACCAA	GCTCTTATCCCAAGGGCTTA
Adgrd2	CCCAGGATGAGGCTGTACTA	GCAGTCAGCACAAACAGAAC

Supplementary Table 2: GO analysis for *mthl1* positively regulated genes

GO function	GO Terms	Gene number	Fold Enrichment	P value	Gene name
Biological Process	detection of chemical stimulus involved in sensory perception of taste	11	29.38	2.41E-12	Gr64d,Gr22b,Gr64a,Gr22d,Gr9a,Gr64b,Gr64c,Ir7a,Gr64e,Gr64f,
Biological Process	sensory perception of sweet taste	6	26.71	4.33E-07	Gr64d,Gr64a,Gr64b,Gr64c,Gr64e,Gr64f,
Biological Process	cell-cell adhesion mediated by cadherin	4	19.08	1.20E-04	Cad88C,CadN2,ft,Cad96Cb
Biological Process	sensory perception of taste	21	17.53	1.17E-18	Gr98d,Gr64d,Gr22b,Gr77a,Gr64a,Gr22d,Gr98b,Gr59d,Gr98c,Gr22a,ppk19,Gr93d,Gr9a,Gr64b,Gr8a,Gr22f,Gr64c,Ir7a,Gr64e,Gr64f,Gr22e,
Biological Process	detection of chemical stimulus involved in sensory perception	21	13.23	1.67E-16	Ir75a,Or33c,Gr64d,Or24a,Gr22b,Gr64a,Gr22d,Or19a,Or49b,Ir92a,Or23a,Or71a,Ir75d,Gr9a,Or94a,Gr64b,Gr8a,Gr64c,Ir7a,Gr64e,Gr64f,
Biological Process	detection of chemical stimulus	33	13.12	2.46E-25	Ir75a,Or33c,Ir52d,Ir54a,Gr64d,Or24a,Gr22b,Ir94a,Ir94b,Ir85a,Gr64a,Ir52b,Ir94e,Gr22d,Or19a,Or49b,Ir92a,Ir52c,Or23a,Or71a,Ir75d,Gr9a,Or94a,Gr64b,Ir94c,Gr8a,Ir60b,Ir68b,Gr64c,Ir7a,Gr64e,Gr64f,Ir62a,
Biological Process	detection of stimulus involved in sensory perception	21	10.7	7.13E-15	Ir75a,Or33c,Gr64d,Or24a,Gr22b,Gr64a,Gr22d,Or19a,Or49b,Ir92a,Or23a,Or71a,Ir75d,Gr9a,Or94a,Gr64b,Gr8a,Gr64c,Ir7a,Gr64e,Gr64f,
Biological Process	detection of stimulus	33	8.99	9.47E-21	Ir75a,Or33c,Ir52d,Ir54a,Gr64d,Or24a,Gr22b,Ir94a,Ir94b,Ir85a,Gr64a,Ir52b,Ir94e,Gr22d,Or19a,Or49b,Ir92a,Ir52c,Or23a,Or71a,Ir75d,Gr9a,Or94a,Gr64b,Ir94c,Gr8a,Ir60b,Ir68b,Gr64c,Ir7a,Gr64e,Gr64f,Ir62a,
Biological Process	sensory perception of chemical stimulus	34	8.29	2.43E-20	Gr98d,Ir75a,Or33c,Gr64d,Or24a,Gr22b,Gr77a,Obp46a,Gr64a,Gr22d,Gr98b,Gr59d,Or19a,Or49b,Ir92a,Gr98c,Or23a,Gr22a,Or71a,Ir75d,ppk19,Gr93d,Gr9a,Or94a,Obp58c,Gr64b,Obp57e,Gr8a,Gr22f,Gr64c,Ir7a,Gr64e,Gr64f,Gr22e,
Biological Process	detection of chemical stimulus involved in sensory perception of smell	9	8.23	3.22E-06	Ir75a,Or33c,Or24a,Or19a,Or49b,Ir92a,Or23a,Or71a,Or94a,
Biological Process	female meiotic nuclear division	6	6.91	3.53E-04	fzy,mei-41,ncd,Grip128,mei-9,vilya,
Biological Process	sensory perception	36	6.43	3.43E-18	Gr98d,Ir75a,Or33c,Gr64d,Or24a,Gr22b,Gr77a,Obp46a,Gr64a,Gr22d,Gr98b,Gr59d,Or19a,Or49b,bw,Ir92a,Gr98c,Or23a,Gr22a,Or71a,Ir75d,ppk19,Gr93d,Gr9a,Or94a,Obp58c,Gr64b,Obp57e,Gr8a,Gr22f,Nmdar2,Gr64c,Ir7a,Gr64e,Gr64f,Gr22e,
Biological Process	reproductive structure development	7	5.77	3.18E-04	InR,br,tin,Dll,puc,nclb,Invadolysin
Biological Process	reproductive system development	7	5.77	3.18E-04	InR,br,tin,Dll,puc,nclb,Invadolysin
Biological Process	sensory perception of smell	10	5.22	3.78E-05	Ir75a,Or33c,Or24a,Or19a,Or49b,Ir92a,Or23a,Or71a,Or94a,Obp57e,
Biological Process	nervous system process	39	4.58	4.87E-15	scb,Gr98d,Abl,Ir75a,Or33c,osk,Gr64d,Or24a,Gr22b,Gr77a,Obp46a,Gr64a,Gr22d,Gr98b,Gr59d,Or19a,Or49b,bw,Ir92a,Gr98c,Or23a,Gr22a,Or71a,Ir75d,ppk19,Gr93d,Gr9a,Or94a,Obp58c,Gr64b,
Biological Process	meiotic nuclear division	11	4.48	5.75E-05	fzy,mei-41,Mer,ncd,sa,thoc5,topi,sa,Grip128,mei-9,vilya,
Biological Process	nuclear chromosome segregation	10	4.39	1.45E-04	mei-41,Mer,ncd,rod,sa,Cap-D2,fbl,mei-9,vilya,Invadolysin,

Biological Process	meiotic cell cycle	14	4.37	7.32E-06	fzy,mei-41,Mer,ncd,sa,Cap-D2,thoc5,topi,cuff,fbl,sa,Grip128,mei-9,vilya,
Biological Process	nuclear division	16	4.31	1.90E-06	fzy,mei-41,Mer,ncd,rod,sa,Cap-D2,tum,thoc5,topi,alphaTub67C,sa,Grip128,mei-9,vilya,Invadolysin,
Biological Process	chromosome segregation	11	4.3	8.20E-05	mei-41,Mer,ncd,rod,sa,Cap-D2,tum,fbl,mei-9,vilya,Invadolysin,
Biological Process	meiotic cell cycle process	13	4.28	1.93E-05	fzy,mei-41,Mer,ncd,sa,Cap-D2,thoc5,topi,cuff,sa,Grip128,mei-9,vilya,
Biological Process	organelle fission	16	4.09	3.55E-06	fzy,mei-41,Mer,ncd,rod,sa,Cap-D2,tum,thoc5,topi,alphaTub67C,sa,Grip128,mei-9,vilya,Invadolysin,
Biological Process	system process	39	4.07	1.83E-13	scb,Gr98d,Abl,Ir75a,Or33c,osk,Gr64d,Or24a,Gr22b,Gr77a,Obp46a,Gr64a,Gr22d,Gr98b,Gr59d,Or19a,Or49b,bw,Ir92a,Gr98c,Or23a,Gr22a,Or71a,Ir75d,ppk19,Gr93d,Gr9a,Or94a,Obp58c,Gr64b,Obp57e,Gr8a,Gr22f,Nmdar2,Gr64c,Ir7a,Gr64e,Gr64f,Gr22e,
Biological Process	response to chemical	61	3.73	2.37E-19	InR,psh,scb,br,dlt,Abl,Ir75a,Or33c,Ir52d,Ir54a,PCNA,kraken,Gr64d,Gclc,Or24a,Cyp6g2,Gr22b,zfh1,Ir94a,gt,CadN2,thoc5,Ir94b,Ir85a,Gr64a,Ir52b,Ir94e,puc,dor,Gr22d,Gr98b,Or19a,Or49b,Ir92a,Ir52c,Or23a,dom,Or71a,nclb,Ir75d,ppk19,Gr9a,Or94a,Gr64b,Ir94c,Obp57e,bnl,Gr8a,Tdc2,Ir60b,Ir68b,Hsp68,Gr64c,Nrt,Ir7a,St4,Gr64e,RhoGAP93B,Gr64f,grn,Ir62a,
Biological Process	positive regulation of transcription by RNA polymerase II	16	3.19	6.60E-05	ase,sage,br,Eip74EF,MED19,Art4,tin,dor,Doc2,cuff,NC2alpha,Doc1,xmas-2,dre4,dimm,grn,
Biological Process	positive regulation of DNA-templated transcription	20	3.1	1.18E-05	ase,sage,br,Eip74EF,MED19,Art4,thoc5,tin,br,dor,topi,Doc2,cuff,nclb,NC2alpha,Doc1,xmas-2,dre4,dimm,grn,
Biological Process	positive regulation of nucleic acid-templated transcription	20	3.1	1.18E-05	ase,sage,br,Eip74EF,MED19,Art4,thoc5,tin,br,dor,topi,Doc2,cuff,nclb,NC2alpha,Doc1,xmas-2,dre4,dimm,grn,
Biological Process	positive regulation of RNA biosynthetic process	20	3.1	1.18E-05	ase,sage,br,Eip74EF,MED19,Art4,thoc5,tin,br,dor,topi,Doc2,cuff,nclb,NC2alpha,Doc1,xmas-2,dre4,dimm,grn,
Biological Process	positive regulation of macromolecule biosynthetic process	22	3.04	5.62E-06	ase,sage,br,Eip74EF,MED19,Art4,thoc5,tin,br,dor,topi,Doc2,cuff,nclb,NC2alpha,Doc1,xmas-2,dre4,dimm,hoip,cutlet,grn,
Biological Process	positive regulation of cellular biosynthetic process	23	2.9	7.08E-06	psh,ase,sage,br,Eip74EF,MED19,Art4,thoc5,tin,br,dor,topi,Doc2,cuff,nclb,NC2alpha,Doc1,xmas-2,dre4,dimm,hoip,cutlet,grn,
Biological Process	positive regulation of biosynthetic process	23	2.9	7.30E-06	psh,ase,sage,br,Eip74EF,MED19,Art4,thoc5,tin,br,dor,topi,Doc2,cuff,nclb,NC2alpha,Doc1,xmas-2,dre4,dimm,hoip,cutlet,grn,
Biological Process	positive regulation of nucleobase-containing compound metabolic process	22	2.87	1.35E-05	ase,sage,br,Eip74EF,MED19,Art4,thoc5,tin,br,dor,topi,Doc2,cuff,nclb,NC2alpha,Doc1,xmas-2,dre4,dimm,hoip,cutlet,grn,
Biological Process	positive regulation of RNA metabolic process	20	2.78	5.15E-05	ase,sage,br,Eip74EF,MED19,Art4,thoc5,tin,br,dor,topi,Doc2,cuff,nclb,NC2alpha,Doc1,xmas-2,dre4,dimm,grn,
Biological Process	cell cycle process	21	2.72	4.48E-05	fzy,mei-41,Mer,ncd,rod,sa,PCNA,Cap-D2,tum,thoc5,topi,alphaTub67C,cuff,sa,Grip128,ALiX,mei-9,Orc1,vilya,Invadolysin,Hsp83,
Biological Process	mitotic cell cycle	16	2.66	4.68E-04	fzy,mei-41,ncd,rod,sa,PCNA,Cap-D2,tum,mh,alphaTub67C,fbl,Grip128,ALiX,Orc1,mi,Invadolysin,

Biological Process	female gamete generation	25	2.65	1.23E-05	InR,fzy,scb,br,Abl,Eip74EF,T-cp1,mei-41,Mer,ncd,osk,PCNA,18w,puc,cuff,fbl,dor,nclb,Grip128,fs(1)Yb,ALiX,mei-9,vilya,mi,Hsp83,
Biological Process	cell cycle	24	2.63	2.18E-05	fzy,mei-41,Mer,ncd,rod,sa,PCNA,Cap-D2,tum,mh,thoc5,topi,alphaTub67C,cuff,fbl,sa,Grip128,ALiX,mei-9,Orc1,vilya,mi,Invadolysin,Hsp83,
Biological Process	response to stimulus	99	2.61	9.89E-22	InR,psh,Rab27,scb,RabX1,Pask,Drsl2,br,dlt,Abl,Ir75a,Cyp6a13,mei-41,RhoGAP15B,Or33c,Mer,RPA2,Tre1,rod,Ir52d,osk,Ir54a,PCNA,kraken,Gr64d,Gclc,Or24a,Cyp6g2,Gr22b,pyx,18w,zfh1,tum,mh,hay,Ir94a,gt,CadN2,thoc5,Ir94b,Ir85a,Gr64a,Ir52b,Ir94e,puc,dor,Gr22d,Gr98b,hdc,Or19a,Idgf1,Or49b,Hsp23,Ir92a,Ir52c,fbl,Or23a,CBP,RhoGAP16F,dom,dor,nclb,Or71a,Ir75d,ppk19,CG14803,TotF,PPO3,Gr9a,Or94a,Gr64b,CCAP,Ir94c,Obp57e,bnl,Gr8a,Drsl3,Tdc2,Ir60b,mthl9,Nmdar2,mei-9,Ir68b,Orc1,Hsp68,ECSIT,Gcn2,Gr64c,Nrt,Ir7a,St4,Gr64e,RhoGAP93B,Cht5,Gr64f,grn,Hsp83,Ir62a,LysX,
Biological Process	cellular process involved in reproduction in multicellular organism	32	2.5	3.47E-06	InR,fzy,scb,br,Abl,Eip74EF,T-cp1,mei-41,Mer,ncd,Tre1,osk,PCNA,18w,zfh1,thoc5,tin,puc,topi,cuff,fbl,sa,dor,nclb,Grip128,fs(1)Yb,ALiX,mei-9,vilya,mi,Invadolysin,Hsp83,
Biological Process	embryo development	19	2.49	3.18E-04	InR,scb,ase,sage,Abl,mei-41,Mer,osk,zfh1,gcm2,gt,tin,puc,alphaTub67C,cuff,Grip128,Doc1,pip,Hsp83,
Biological Process	positive regulation of nitrogen compound metabolic process	28	2.47	1.88E-05	psh,fzy,ase,sage,br,Eip74EF,Mer,MED19,Art4,thoc5,tin,br,dor,topi,Doc2,cuff,nclb,NC2alpha,Doc1,xmas-2,pip,bnl,dre4,vilya,dimm,hoip,cutlet,grn,
Biological Process	tissue development	37	2.46	5.72E-07	InR,Sox15,scb,ase,mey,sage,br,hll,dlt,Abl,T-cp1,mei-41,RhoGAP15B,hdc,PCNA,Gclc,18w,zfh1,CadN2,tin,br,puc,Idgf1,Doc2,fbl,nyo,RhoGAP16F,dom,dor,nclb,Doc1,ft,bnl,dimm,hoip,Invadolysin,grn,
Biological Process	regulation of multicellular organismal process	21	2.41	3.42E-04	InR,scb,ase,mey,hll,Abl,Mer,hdc,gcm2,gt,puc,bchs,nyo,dom,cue,ft,CCAP,pip,bnl,b6,Hsp83,
Biological Process	positive regulation of cellular metabolic process	28	2.39	2.65E-05	InR,psh,fzy,ase,sage,br,Eip74EF,Mer,MED19,Art4,thoc5,tin,br,dor,topi,Doc2,cuff,nclb,NC2alpha,Doc1,xmas-2,bnl,dre4,vilya,dimm,hoip,cutlet,grn,
Biological Process	positive regulation of macromolecule metabolic process	29	2.35	2.39E-05	psh,fzy,ase,sage,br,Eip74EF,Mer,MED19,Art4,thoc5,tin,br,puc,dor,topi,Doc2,cuff,nclb,NC2alpha,Doc1,xmas-2,pip,bnl,dre4,vilya,dimm,hoip,cutlet,grn,

Biological Process	multicellular organismal process	116	2.33	2.73E-22	InR,Sox15,fzy,scb,ase,Gr98d,sage,br,dlt,Abl,Ir75a,Eip74EF,T-cp1,crm,Cad88C,mei-41,RhoGAP15B,Or33c,Mer,ncd,Tre1,twz,Ir52d,sa,osk,hdc,PCNA,kraken,Gr64d,Gclc,Cap-D2,Or24a,Gr22b,18w,zfh1,tum,gcm2,Gr77a,Obp46a,gt,CadN2,thoc5,Spt,Gr64a,tin,br,puc,Gr22d,topi,Gr98b,Gr59d,Or19a,Idgf1,alphaTub67C,Or49b,bchs,Doc2,bw,cuff,TwdlV,Ir92a,Gr98c,Ir52c,fbl,Or23a,exp,RhoGAP16F,Gr22a,natalisin,sa,dom,dor,nclb,Or71a,Ir75d,Grip128,ppk19,fs(1)Yb,Nmnat,ALiX,Gr93d,Doc1,Gr9a,Or94a,Obp58c,Gr64b,ft,Cpr72Eb,pip,Obp57e,bnl,Gr8a,Tdc2,Gr22f,mthl9,Nmdar2,mei-9,vilya,Hsp68,dimm,Gr64c,mi,Cad96Cb,Nrt,Ir7a,Gr64e,RhoGAP93B,Cht5,Invadolysin,tamo,Gr64f,grn,Acp65Aa,Hsp83,Gr22e,Cpr97Ea,
Biological Process	gamete generation	32	2.32	1.00E-05	InR,fzy,scb,br,Abl,Eip74EF,T-cp1,mei-41,Mer,ncd,Tre1,osk,PCNA,18w,zfh1,thoc5,tin,puc,topi,cuff,fbl,sa,dor,nclb,Grip128,fs(1)Yb,ALiX,mei-9,vilya,mi,Invadolysin,Hsp83,
Biological Process	animal organ development	40	2.3	9.54E-07	InR,Sox15,scb,ase,sage,br,dlt,Abl,Eip74EF,mei-41,RhoGAP15B,Mer,hdc,Gclc,Cap-D2,18w,zfh1,tum,gcm2,gt,CadN2,tin,br,puc,Idgf1,bchs,Doc2,fbl,exp,RhoGAP16F,dom,nclb,Doc1,ft,bnl,dimm,mi,hoip,Invadolysin,grn,
Biological Process	epithelium development	32	2.3	1.82E-05	InR,Sox15,scb,ase,mey,br,dlt,Abl,T-cp1,mei-41,RhoGAP15B,hdc,PCNA,Gclc,18w,CadN2,tin,br,puc,Idgf1,Doc2,fbl,nyo,RhoGAP16F,dom,dor,nclb,Doc1,ft,bnl,dimm,Invadolysin,
Biological Process	reproductive process	41	2.29	7.40E-07	InR,fzy,scb,br,Abl,Eip74EF,T-cp1,mei-41,Mer,ncd,Tre1,twz,Ir52d,sa,osk,PCNA,Cap-D2,18w,zfh1,thoc5,tin,br,puc,topi,alphaTub67C,cuff,Ir52c,fbl,natalisin,sa,dor,nclb,Grip128,fs(1)Yb,ALiX,Tdc2,mei-9,vilya,mi,Invadolysin,Hsp83,
Biological Process	multicellular organismal reproductive process	32	2.27	2.06E-05	InR,fzy,scb,br,Abl,Eip74EF,T-cp1,mei-41,Mer,ncd,Tre1,osk,PCNA,18w,zfh1,thoc5,tin,puc,topi,cuff,fbl,sa,dor,nclb,Grip128,fs(1)Yb,ALiX,mei-9,vilya,mi,Invadolysin,Hsp83,
Biological Process	multicellular organism reproduction	37	2.24	4.27E-06	InR,fzy,scb,br,Abl,Eip74EF,T-cp1,mei-41,Mer,ncd,Tre1,twz,Ir52d,osk,PCNA,18w,zfh1,thoc5,tin,puc,topi,cuff,Ir52c,fbl,natalisin,sa,dor,nclb,Grip128,fs(1)Yb,ALiX,Tdc2,mei-9,vilya,mi,Invadolysin,Hsp83,
Biological Process	positive regulation of metabolic process	30	2.19	6.38E-05	InR,psh,fzy,ase,sage,br,Eip74EF,Mer,MED19,Art4,thoc5,tin,br,puc,dor,topi,Doc2,cuff,nclb,NC2alpha,Doc1,xmas-2,pip,bnl,dre4,vilya,dimm,hoip,cutlet,grn,
Biological Process	cell differentiation	47	2	4.44E-06	InR,Sox15,scb,ase,mey,br,Abl,Eip74EF,T-cp1,mei-41,Mer,Tre1,sa,osk,hdc,PCNA,Gclc,18w,zfh1,tum,gcm2,gt,CadN2,tin,puc,bchs,Doc2,cuff,fbl,nyo,sa,dom,dor,nclb,Grip128,fs(1)Yb,ALiX,Doc1,ft,bnl,mei-9,dimm,mi,Nrt,RhoGAP93B,grn,Hsp83,
Biological Process	cellular developmental process	47	1.99	4.88E-06	InR,Sox15,scb,ase,mey,br,Abl,Eip74EF,T-cp1,mei-41,Mer,Tre1,sa,osk,hdc,PCNA,Gclc,18w,zfh1,tum,gcm2,gt,CadN2,tin,puc,bchs,Doc2,cuff,fbl,nyo,sa,dom,dor,nclb,Grip128,fs(1)Yb,ALiX,Doc1,ft,bnl,mei-9,dimm,mi,Nrt,RhoGAP93B,grn,Hsp83,

Biological Process	sexual reproduction	34	1.98	1.97E-04	InR,fzy,scb,br,Abl,Eip74EF,T-cp1,mei-41,Mer,ncd,Tre1,osk,PCNA,18w,zfh1,thoc5,tin,puc,topi,alphaTub67C,cuff,fbl,sa,dor,nclb,Grip128,fs(1)Yb,ALiX,mei-9,vilya,mi,Invadolysin,Peritrophin-A,Hsp83,
Biological Process	reproduction	42	1.96	2.79E-05	InR,fzy,scb,br,Abl,Eip74EF,T-cp1,mei-41,Mer,ncd,Tre1,twz,Ir52d,sa,osk,PCNA,Cap-D2,18w,zfh1,thoc5,tin,br,puc,topi,alphaTub67C,cuff,Ir52c,fbl,natalisin,sa,dor,nclb,Grip128,fs(1)Yb,ALiX,Tdc2,mei-9,vilya,mi,Invadolysin,Peritrophin-A,Hsp83,
Biological Process	response to stress	33	1.92	3.46E-04	InR,psh,scb,Drsl2,Cyp6a13,mei-41,RPA2,PCNA,pyx,18w,tum,mh,hay,thoc5,puc,Idgf1,Hsp23,fbl,CBP,dor,ppk19,CG14803,TotF,PPO3,Drsl3,mthl9,mei-9,Hsp68,ECSIT,Gcn2,Cht5,Hsp83,LysX,
Biological Process	cell development	37	1.9	1.79E-04	InR,scb,mey,br,Abl,Eip74EF,T-cp1,Mer,Tre1,sa,osk,hdc,PCNA,Gclc,18w,zfh1,tum,gcm2,gt,CadN2,puc,bchs,cuff,fbl,nyo,sa,dom,dor,nclb,Grip128,fs(1)Yb,ALiX,mi,Nrt,RhoGAP93B,grn,Hsp83,
Biological Process	system development	38	1.89	1.37E-04	InR,scb,ase,sage,br,Abl,Eip74EF,Cad88C,Mer,sa,hdc,Gclc,Cap-D2,18w,zfh1,tum,gcm2,gt,CadN2,tin,br,puc,alphaTub67C,bchs,Doc2,exp,dom,nclb,ppk19,Doc1,ft,bnl,dimm,Cad96Cb,Nrt,RhoGAP93B,Invadolysin,grn,
Biological Process	anatomical structure morphogenesis	41	1.87	9.83E-05	InR,Sox15,scb,ase,mey,sage,br,dlt,Abl,Eip74EF,mei-41,RhoGAP15B,Mer,osk,hdc,PCNA,Gclc,18w,zfh1,tum,gcm2,gt,CadN2,Spt,tin,br,puc,cuff,fbl,exp,nyo,RhoGAP16F,dom,nclb,ft,bnl,mi,Nrt,hoip,RhoGAP93B,grn,
Biological Process	anatomical structure development	70	1.85	1.75E-07	InR,Sox15,scb,ase,sage,mey,br,hll,dlt,Abl,Eip74EF,T-cp1,crm,Cad88C,mei-41,RhoGAP15B,Mer,Tre1,sa,osk,hdc,PCNA,Gclc,Cap-D2,18w,zfh1,tum,gcm2,gt,CadN2,Spt,tin,br,puc,Idgf1,alphaTub67C,bchs,Doc2,cuff,TwdlV,fbl,exp,nyo,RhoGAP16F,sa,dom,dor,nclb,Grip128,ppk19,fs(1)Yb,ALiX,Doc1,Cpr72Eb,ft,pip,bnl,dimm,mi,Cad96Cb,Nrt,hoip,RhoGAP93B,Cht5,Invadolysin,tamo,grn,Acp65Aa,Hsp83,Cpr97Ea,
Biological Process	developmental process	73	1.83	1.15E-07	InR,Sox15,fzy,scb,ase,sage,mey,br,hll,dlt,Abl,Eip74EF,T-cp1,crm,Cad88C,mei-41,RhoGAP15B,Mer,Tre1,sa,osk,hdc,PCNA,Gclc,Cap-D2,18w,zfh1,tum,gcm2,gt,CadN2,Spt,tin,br,puc,topi,Idgf1,alphaTub67C,bchs,Doc2,cuff,TwdlV,fbl,exp,nyo,RhoGAP16F,sa,dom,dor,nclb,Grip128,ppk19,fs(1)Yb,ALiX,Doc1,Cpr72Eb,ft,pip,bnl,mei-9,dimm,mi,Cad96Cb,Nrt,hoip,RhoGAP93B,Cht5,Invadolysin,tamo,grn,Acp65Aa,Hsp83,Cpr97Ea
Biological Process	cellular response to stimulus	41	1.83	1.75E-04	InR,Sox15,scb,ase,mey,sage,br,dlt,Abl,Eip74EF,mei-41,RhoGAP15B,Mer,osk,hdc,PCNA,Gclc,18w,zfh1,tum,gcm2,gt,CadN2,Spt,tin,br,puc,cuff,fbl,exp,nyo,RhoGAP16F,dom,nclb,ft,bnl,mi,Nrt,hoip,RhoGAP93B,grn,

Biological Process	multicellular organism development	58	1.82	4.16E-06	InR,Sox15,scb,ase,sage,br,dlt,Abl,Eip74EF,crm,Cad88C,mei-41,RhoGAP15B,Mer,sa,osk,hdc,Gclc,Cap-D2,18w,zfh1,tum,gcm2,gt,CadN2,Spt,tin,br,puc,Idgf1,alphaTub67C,bchs,Doc2,cuff,TwdlV,fbl,exp,RhoGAP16F,dom,nclb,Grip128,ppk19,Doc1,Cpr72Eb,ft,pip,bnl,dimm,Cad96Cb,Nrt,RhoGAP93B,Cht5,Invadolysin,tamo,grn,Acp65Aa,Hsp83,Cpr97Ea,
Biological Process	positive regulation of cellular process	42	1.79	2.35E-04	InR,psh,fzy,Rab27,ase,sage,br,Abl,Eip74EF,Mer,twz,MED19,nopo,Art4,thoc5,tin,br,puc,dor,topi,Doc2,cuff,Ir92a,RhoGEF4,dor,nclb,NC2alpha,ALiX,Doc1,xmas-2,ft,pip,bnl,dre4,vilya,dimm,mi,hoip,cutlet,Invadolysin,grn,Hsp83,
Biological Process	positive regulation of biological process	46	1.75	1.42E-04	InR,psh,fzy,Rab27,ase,sage,br,hll,Abl,Eip74EF,Mer,twz,MED19,nopo,Art4,thoc5,tin,br,puc,dor,topi,bchs,Doc2,cuff,Ir92a,RhoGEF4,dor,nclb,NC2alpha,ALiX,cue,Doc1,xmas-2,ft,CCAP,pip,bnl,dre4,vilya,dimm,mi,hoip,cutlet,Invadolysin,grn,Hsp83,
Biological Process	regulation of cellular process	83	1.5	3.53E-05	InR,psh,Sox15,fzy,Rab27,scb,ase,sage,RabX1,Pask,br,dlt,Abl,Ir75a,Eip74EF,crm,mei-41,RhoGAP15B,Mer,MED9,ncd,Tre1,twz,rod,osk,hdc,PCNA,MED19,18w,zfh1,tum,nopo,Hers,gcm2,Art4,gt,thoc5,Rph,tin,br,puc,dor,topi,Idgf1,bchs,Doc2,cuff,Ir92a,fbl,exp,RhoGEF4,RhoGAP16F,dom,pgant3,dor,nclb,NC2alpha,Ns3,fs(1)Yb,Nmnat,ALiX,Doc1,xmas-2,ft,CCAP,pip,bnl,dre4,mthl9,Nmdar2,Orc1,vilya,dimm,Gcn2,mi,b6,hoip,cutlet,RhoGAP93B,Invadolysin,tamo,grn,Hsp83,
Biological Process	regulation of biological process	89	1.47	2.90E-05	InR,psh,Sox15,fzy,Rab27,scb,ase,sage,mey,RabX1,Pask,br,hll,dlt,Abl,Ir75a,Eip74EF,crm,mei-41,RhoGAP15B,Mer,MED9,ncd,Tre1,twz,rod,osk,hdc,PCNA,MED19,18w,zfh1,tum,nopo,Hers,gcm2,Art4,gt,thoc5,Rph,tin,br,puc,dor,topi,Idgf1,bchs,Doc2,cuff,Ir92a,fbl,exp,RhoGEF4,nyo,RhoGAP16F,sa,dom,pgant3,dor,nclb,NC2alpha,Ns3,fs(1)Yb,Nmnat,ALiX,cue,Doc1,xmas-2,ft,CCAP,pip,bnl,Teh4,dre4,mthl9,Nmdar2,Orc1,vilya,dimm,Gcn2,mi,b6,hoip,cutlet,RhoGAP93B,Invadolysin,tamo,grn,Hsp83,
Biological Process	biological regulation	92	1.37	3.18E-04	InR,psh,Sox15,fzy,Rab27,scb,ase,sage,mey,RabX1,Pask,br,hll,dlt,Abl,Ir75a,Eip74EF,Best1,crm,mei-41,RhoGAP15B,Mer,MED9,ncd,Tre1,twz,rod,osk,hdc,PCNA,MED19,18w,zfh1,tum,nopo,Hers,gcm2,Art4,gt,thoc5,Rph,tin,br,puc,dor,topi,Idgf1,bchs,Doc2,cuff,Ir92a,fbl,exp,RhoGEF4,nyo,RhoGAP16F,sa,dom,pgant3,dor,nclb,NC2alpha,Ns3,fs(1)Yb,Nmnat,ALiX,cue,Doc1,xmas-2,ft,CCAP,pip,bnl,Teh4,dre4,mthl9,Nmdar2,Orc1,vilya,dimm,ECSIT,Gcn2,mi,b6,hoip,cutlet,rho-4,RhoGAP93B,Invadolysin,tamo,grn,Hsp83,
Molecular Function	sweet taste receptor activity	15	37.09	1.4E-17	Gr98d,Gr64d,Gr22b,Gr64a,Gr22d,Gr98b,Gr59d,Gr98c,Gr22a,Gr64b,Gr22f,Gr64c,Gr64e,Gr64f,Gr22e,
Molecular Function	taste receptor activity	19	21.14	2.9E-18	Gr98d,Gr64d,Gr22b,Gr77a,Gr64a,Gr22d,Gr98b,Gr59d,Gr98c,Gr22a,Gr93d,Gr9a,Gr64b,Gr22f,Gr64c,Gr64e,Gr64f,Gr22e,
Molecular Function	olfactory receptor activity	9	8.58	2.4E-06	Ir75a,Or33c,Or24a,Or19a,Or49b,Ir92a,Or23a,Or71a,Or94a,

Molecular Function	ligand-gated ion channel activity	18	8.4	2.5E-11	Ir75a,Ir52d,Ir54a,Ir94a,Ir94b,Ir85a,Ir52b,Ir94e,Ir92a,Ir52c,Ir75d,ppk19,ppk21,Ir94c,Ir60b,Nmdar2,Ir68b,Ir52a,Ir7a,Ir62a,
Molecular Function	ligand-gated channel activity	18	8.4	2.5E-11	Ir75a,Ir52d,Ir54a,Ir94a,Ir94b,Ir85a,Ir52b,Ir94e,Ir92a,Ir52c,Ir75d,ppk19,ppk21,Ir94c,Ir60b,Nmdar2,Ir68b,Ir52a,Ir7a,Ir62a,
Molecular Function	transmembrane signaling receptor activity	33	6.5	7.4E-17	InR,Gr98d,Ir75a,Or33c,Tre1,Gr64d,Or24a,Gr22b,Gr77a,Gr64a,Gr22d,Gr98b,Gr59d,Or19a,Or49b,Ir92a,Gr98c,Or23a,Gr22a,Or71a,Ror,Gr93d,Gr9a,Or94a,Gr64b,Gr22f,mthl9,Nmdar2,Gr64c,Gr64e,Gr64f,Gr22e,
Molecular Function	gated channel activity	19	6.28	6.8E-10	Ir75a,Best1,Ir52d,Ir54a,Ir94a,Ir94b,Ir85a,Ir52b,Ir94e,Ir92a,Ir52c,Ir75d,ppk19,ppk21,Ir94c,Ir60b,Nmdar2,Ir68b,Ir52a,Ir7a,Ir62a,
Molecular Function	odorant binding	10	6.02	1.2E-05	Or33c,Or24a,Obp46a,Or19a,Or49b,Or23a,Or71a,Or94a,Obp58c,Obp57e,
Molecular Function	signaling receptor activity	35	5.76	2.5E-16	InR,Gr98d,Ir75a,Or33c,Tre1,Gr64d,Or24a,Gr22b,18w,Gr77a,Gr64a,Gr22d,Gr98b,Gr59d,Or19a,Or49b,Ir92a,Gr98c,Or23a,Gr22a,Or71a,Ir75d,Gr93d,Gr9a,Or94a,Gr64b,Gr8a,Gr22f,mthl9,Nmdar2,Gr64c,Nrt,Gr64e,Gr64f,Gr22e,
Molecular Function	molecular transducer activity	35	5.76	2.5E-16	InR,Gr98d,Ir75a,Or33c,Tre1,Gr64d,Or24a,Gr22b,18w,Gr77a,Gr64a,Gr22d,Gr98b,Gr59d,Or19a,Or49b,Ir92a,Gr98c,Or23a,Gr22a,Or71a,Ir75d,Gr93d,Gr9a,Or94a,Gr64b,Gr8a,Gr22f,mthl9,Nmdar2,Gr64c,Nrt,Gr64e,Gr64f,Gr22e,
Molecular Function	ion channel activity	20	5.16	5.6E-09	Ir75a,Best1,Ir52d,Ir54a,pyx,Ir94a,Ir94b,Ir85a,Ir52b,Ir94e,Ir92a,Ir52c,Ir75d,ppk19,ppk21,Ir94c,Ir60b,Nmdar2,Ir68b,Ir52a,Ir7a,Ir62a,
Molecular Function	channel activity	20	4.87	1.4E-08	Ir75a,Best1,Ir52d,Ir54a,pyx,Ir94a,Ir94b,Ir85a,Ir52b,Ir94e,Ir92a,Ir52c,Ir75d,ppk19,ppk21,Ir94c,Ir60b,Nmdar2,Ir68b,Ir52a,Ir7a,Ir62a,
Molecular Function	passive transmembrane transporter activity	20	4.87	1.4E-08	Ir75a,Best1,Ir52d,Ir54a,pyx,Ir94a,Ir94b,Ir85a,Ir52b,Ir94e,Ir92a,Ir52c,Ir75d,ppk19,ppk21,Ir94c,Ir60b,Nmdar2,Ir68b,Ir52a,Ir7a,Ir62a,
Molecular Function	ion transmembrane transporter activity	24	3.31	4.9E-07	Ir75a,Best1,Ir52d,Ir54a,pyx,Ir94a,Ir94b,Ir85a,Ir52b,Ir94e,Zip48C,Tpc2,CG17119,Ir92a,Ir52c,Ir75d,ppk19,Ir94c,l(2)03659,Ir60b,Nmdar2,Ir68b,Ir7a,Ir62a,
Molecular Function	inorganic molecular entity transmembrane transporter activity	21	3.27	3.2E-06	Ir75a,Best1,Ir52d,Ir54a,pyx,Ir94a,Ir94b,Ir85a,Ir52b,Ir94e,Zip48C,Ir92a,Ir52c,Ir75d,ppk19,Ir94c,Ir60b,Nmdar2,Ir68b,Ir7a,Ir62a,
Molecular Function	DNA binding	29	2.04	0.00029	Sox15,ase,sage,br,Eip74EF,crm,RPA2,PCNA,zfh1,mh,Hers,hay,gcm2,gt,tin,br,topi,RPA2,Doc2,cuff,RPA2,nclb,NC2alpha,Doc1,mei-9,Orc1,dimm,cutlet,grn,
Cellular Component	cortical microtubule cytoskeleton	3	50.08	0.00011	ncd,tum,alphaTub67C
Cellular Component	dendrite	22	7.06	3.4E-12	Gr98d,Ir75a,Or33c,hdc,Or24a,Gr22b,Gr77a,Gr22d,Gr98b,Gr59d,Or19a,Or49b,Gr98c,Or23a,Gr22a,Or71a,Gr93d,Gr9a,Or94a,Gr8a,Gr22f,Gr22e,
Cellular Component	dendritic tree	22	7.06	3.4E-12	Gr98d,Ir75a,Or33c,hdc,Or24a,Gr22b,Gr77a,Gr22d,Gr98b,Gr59d,Or19a,Or49b,Gr98c,Or23a,Gr22a,Or71a,Gr93d,Gr9a,Or94a,Gr8a,Gr22f,Gr22e,
Cellular Component	dendrite membrane	7	6.98	0.00011	Or33c,Or24a,Or19a,Or49b,Or23a,Or71a,Or94a,
Cellular Component	neuronal cell body	16	6.68	7.1E-09	Gr98d,br,Gr22b,Gr77a,Gr22d,Gr98b...Gr59d,Gr98c,Or23a,Gr22a,Nmnat,Gr93d,Gr9a,Gr22f,Gr22e,

Cellular Component	cell body	17	6.6	2.8E-09	Gr98d,br,Ir75a,Gr22b,Gr77a,Gr22d,Gr98b,Gr59d,Gr98c,Or23a,Gr22a,Nmnat,Gr93d,Gr9a,Gr8a,Gr22f,Gr22e,
Cellular Component	perinuclear region of cytoplasm	7	6.07	0.00024	ncd,hdc,Gclc,tum,alphaTub67C,cuff,Hsp83,
Cellular Component	somatodendritic compartment	24	5.64	2.7E-11	Gr98d,br,Ir75a,Or33c,hdc,Or24a...Gr22b,Gr77a,Gr22d,Gr98b,Gr59d,Or19a,Or49b,Gr98c,Or23a,Gr22a,Or71a,Nmnat,Gr93d,Gr9a,Or94a,Gr22f,Gr22e,
Cellular Component	neuron projection membrane	7	4.97	0.00073	Or33c,Or24a,Or19a,Or49b,Or23a,Or71a,Or94a,
Cellular Component	axon	17	4.49	5.3E-07	InR,Gr98d,Abl,hdc,Gr22b,Burs...Gr77a,Gr22d,Gr98b,Gr59d,Gr98c,Gr22a,Gr93d,Gr9a,Gr22f,Nrt,Gr22e,
Cellular Component	neuron projection	25	3.46	1.2E-07	InR,Gr98d,Abl,Ir75a,Or33c,hdc,Or24a,Gr22b,Gr77a,Gr22d,Gr98b,Gr59d,Or19a,Or49b,Gr98c,Or23a,Gr22a,Or71a,Gr93d,Gr9a,Or94a,Gr8a,Gr22f,Nrt,Gr22e,
Cellular Component	integral component of membrane	67	2.94	3.3E-16	InR,scb,Gr98d,mey,Ir75a,Best1,Cad88C,Or33c,Tre1,Ir52d,Ir54a,Gr64d,Or24a,Gr22b,pyx,Tsp42Ec,Ir94a,Gr77a,CadN2,Ir94b,Ir85a,Rph,Gr64a,Ir52b,Ir94e,Gr22d,Gr98b,Gr59d,Tpc2,Or19a,Or49b,bw,Ir92a,Gr98c,Ir52c,Or23a,nyo,Gr22a,Or71a,Ir75d,Osi20,ppk19,Gr93d,Gr9a,Or94a,Gr64b,ft,pip,Ir94c,l(2)03659,Gr8a,Gr22f,Ir60b,Teh4,mthl9,Nmdar2,Ir68b,Gr64c,Tsp42En,Cad96Cb,Ir7a,Gr64e,rho-4,Gr64f,Gr22e,Ir62a,Tsp42Ed,
Cellular Component	intrinsic component of membrane	67	2.88	8.4E-16	InR,scb,Gr98d,mey,Ir75a,Best1,Cad88C,Or33c,Tre1,Ir52d,Ir54a,Gr64d,Or24a,Gr22b,pyx,Tsp42Ec,Ir94a,Gr77a,CadN2,Ir94b,Ir85a,Rph,Gr64a,Ir52b,Ir94e,Gr22d,Gr98b,Gr59d,Tpc2,Or19a,Or49b,bw,Ir92a,Gr98c,Ir52c,Or23a,nyo,Gr22a,Or71a,Ir75d,Osi20,ppk19,Gr93d,Gr9a,Or94a,Gr64b,ft,pip,Ir94c,l(2)03659,Gr8a,Gr22f,Ir60b,Teh4,mthl9,Nmdar2,Ir68b,Gr64c,Tsp42En,Cad96Cb,Ir7a,Gr64e,rho-4,Gr64f,Gr22e,Ir62a,Tsp42Ed,
Cellular Component	plasma membrane bounded cell projection	29	2.66	2.3E-06	InR,Gr98d,Abl,Ir75a,Or33c,hdc,Or24a,Gr22b,Gr77a,Gr22d,Gr98b,Gr59d,Or19a,Or49b,Gr98c,Or23a,Gr22a,BBS8,Or71a,Gr93d,Gr9a,Or94a,bnl,Gr8a,Gr22f,Mks1,Nrt,cep290,Gr22e,
Cellular Component	cell projection	29	2.63	2.7E-06	InR,Gr98d,Abl,Ir75a,Or33c,hdc,Or24a,Gr22b,Gr77a,Gr22d,Gr98b,Gr59d,Or19a,Or49b,Gr98c,Or23a,Gr22a,BBS8,Or71a,Gr93d,Gr9a,Or94a,bnl,Gr8a,Gr22f,Mks1,Nrt,cep290,Gr22e,
Cellular Component	chromosome	21	2.34	0.00043	fzy,sage,br,RPA2,rod,sa,PCNA,Cap-D2,nopo,thoc5,topi,RPA2,dom,nclb,CG14803,pip,dre4,Orc1,vilya,cutlet,Hsp83,
Cellular Component	membrane	83	1.85	3E-09	InR,scb,Gr98d,mey,Abl,Ir75a,Best1,Cad88C,Or33c,Mer,Tre1,Ir52d,Ir54a,Gr64d,Or24a,Gr22b,pyx,18w,tum,Tsp42Ec,Ir94a,Gr77a,CadN2,Ir94b,Ir85a,Spt,Rph,Gr64a,Ir52b,Ir94e,Gr22d,Gr98b,Zip48C,Gr59d,Tpc2,CG17119,Or19a,alphaTub67C,Or49b,bw,Ir92a,Gr98c,Ir52c,fbl,Or23a,nyo,Gr22a,dor,Or71a,Ir75d,Osi20,ppk19,Gr93d,Gr9a,Or94a,Gr64b,ft,pip,Ir94c,l(2)03659,Gr8a,Gr22f,Ir60b,Teh4,mthl9,Nmdar2,Ir68b,Hsp68,Corin,Gr64c,Tsp42En,Cad96Cb,Nrt,Ir7a,Gr64e,rho-4,Invadolysin, Gr64f, Gr22e,Sr-CIII,Hsp83,Ir62a,Tsp42Ed,

Cellular Component	cellular anatomical entity	191	1.37	1.9E-17	InR,psh,Sox15,fzy,Rab27,scb,ase,frm,Gr98d,mey,sage,RabX1,Pask,Drsl2,br,dlt,Abl,Ir75a,Eip74EF,Best1,T-cp1,crm,Cad88C,mei-41, RhoGAP15B,GstD3,Or33c,Mer,MED9,ncd,RPA2,Tre1,rod,Ir52d,sa,osk,hdc,Ir54a,PCNA,Gr64d,Gclc,Cap-D2,Or24a,Gr22b,pyx,18w,MED19, zfh1,tum,mh,nopo,Hers,hay,Tsp42Ec,gcm2,Art4,Mur2B,Ir94a,Gr77a,Obp46a,gt,CadN2,thoc5,Ir94b,Ir85a,Spt,Rph,Snup,Gr64a,tin,Ir52b,Ir94e,br,puc,dor,Gr22d,topi,Gr98b,Zip48C,Gr59d,Tpc2,hdc, CG17119,Or19a,Idgf1,l(2)k05911,alphaTub67C, RPA2,Or49b,bchs,Doc2,bw,cuff,TwdlV,Hsp23,Ir92a,Gr98c,CG9346,Ir52c,fbl,Or23a,yellow-g2, exp,CBP,RhoGEF4,nyo,RhoGAP16F,Gr22a,BBS8,RPA2,sa,dom,dor,pgant3,nclb,Or71a,Ir75d,NC2alpha,Grip128,Osi20,ppk19,Ns3,fs(1)Yb,CG9143,Nmnat,CG14803,TotF,ALiX,Gr93d,PPO3,Doc1,Gr9a,Or94a,Obp58c,Gr64b,xmas-2,ft, Cpr72Eb,CCAP,pip,Ir94c,Obp57e,l(2)03659,bnl, Gr8a,Drsl3,Tdc2,obst-G,Gr22f,Ir60b,Teh4, dre4,mthl9,Nmdar2,mei-9,Ir68b,Orc1,vilya,Hsp68, dimm,ECSIT,Corin,Gcn2,Gr64c,Tsp42En,Mks1, Cad96Cb,Nrt,Ir7a,b6,hoip,St4,cutlet,Gr64e,rho-4,RhoGAP93B,Cht5, Invadolysin,tamo,Gr64f, cep290,Peritrophin-A,Acp65Aa,Sr-CIII,Hsp83, Gr22e,Ir62a,Tsp42Ed,bcn92, LysX,Cpr97Ea,

Supplementary Table 3: GO analysis for *mthl1* positively regulated genes

GO function	GO Terms	Gene number	Fold Enrichment	P value	Gene name
Biological Process	positive regulation of pathway-restricted SMAD protein phosphorylation	3	26.68	0.00043	dpp,scw,daw,
Biological Process	regulation of epithelial cell migration, open tracheal system	4	24.9	5.4E-05	hh,sli,Stam,robo2,
Biological Process	regulation of pathway-restricted SMAD protein phosphorylation	3	23.35	0.00059	dpp,scw,daw,
Biological Process	body morphogenesis	9	20.01	4.4E-09	TwdlK,dpp,TwdlS,TwdlN,TwdlX,TwdlJ,TwdlO,TwdlR,Ndg,
Biological Process	basement membrane organization	5	14.82	4.7E-05	lama,Col4a1,prc,vkg,Ndg
Biological Process	regulation of epithelial cell migration	8	9.58	4.3E-06	hh,sli,Diap1,Sin3A,tai,Stam,robo2,slbo,
Biological Process	epithelial cell migration, open tracheal system	5	8.65	0.00045	dpp,hh,sli,Stam,dysf
Biological Process	heart morphogenesis	5	8.41	0.0005	hh,sli,spdo,robo2,prc
Biological Process	RNA export from nucleus	6	7.32	0.00027	Nup154,Mat89Ba,Nup153,tpr,Ref1,Hel25E
Biological Process	regulation of cell migration	9	6.3	2.4E-05	hh,sli,lama,Diap1,Sin3A,tai,Stam,robo2,slbo,
Biological Process	regulation of cell motility	9	6.02	3.4E-05	hh,sli,lama,Diap1,Sin3A,tai,Stam,robo2,slbo,
Biological Process	cell fate specification	8	5.86	0.00011	dpp,hh,Brd,rho,Tom,daw,robo2,ocm
Biological Process	heart development	9	5.78	4.6E-05	dpp,hh,sli,lama,srp,spdo,Col4a1,robo2,prc
Biological Process	circulatory system development	9	5.78	4.6E-05	dpp,hh,sli,lama,srp,spdo,Col4a1,robo2,prc
Biological Process	sex differentiation	9	5.72	4.9E-05	foi,dpp,hh,sli,dmrt11E,scw,Yp1,robo2,ken
Biological Process	mesoderm development	9	5.72	4.9E-05	foi,dpp,hh,sli,lama,srp,scw,bib,E(spl)m8-HLH
Biological Process	chitin-based cuticle development	18	5.66	9.5E-09	TwdlK,TwdlH,Vajk3,TwdlE,Cpr49Af,TwdlS,Cpr72Ea,Cpr65Ec,Cht2,TwdlC,TwdlN,Cpr78Ca,TwdlX,TwdlJ,Cpr78E,TwdlO,Cpr78Cc,TwdlR
Biological Process	cuticle development	21	5.64	5.6E-10	TwdlK,TwdlH,Vajk3,TwdlE,Cpr49Af,Cpr76Bb,Cpr72Ea,Cpr65Ec,ovo,Cht2,TwdlC,y,TwdlN,Cpr78Ca,TwdlX,TwdlJ,Cpr78E,TwdlO,Cpr78Cc,Lcp9,
Biological Process	reproductive structure development	7	5.38	0.00048	foi,hh,sli,scw,Col4a1,robo2,ken
Biological Process	reproductive system development	7	5.38	0.00048	foi,hh,sli,scw,Col4a1,robo2,ken
Biological Process	regulation of locomotion	10	5.02	5.3E-05	hh,sli,lama,Diap1,Sin3A,Vang,tai,Stam,robo2,slbo
Biological Process	digestive tract development	9	5	0.00013	dpp,hh,sli,srp,scw,trh,rho,Col4a1,vkg
Biological Process	digestive system development	9	5	0.00013	dpp,hh,sli,srp,scw,trh,rho,Col4a1,vkg
Biological Process	epigenetic regulation of gene expression	9	4.96	0.00014	Pc,ph-p,tara,escl,Nup153,Su(var)2-HP2,tpr,ocm,aub
Biological Process	external encapsulating structure organization	11	4.66	4.2E-05	Ppn,dpp,lama,lama,Cp16,Col4a1,Femcoat,prc,vkg,aub,Ndg
Biological Process	epithelial cell migration	10	4.45	0.00014	dpp,hh,sli,Diap1,Sin3A,Vang,tai,Stam,dysf,slbo,
Biological Process	epithelium migration	10	4.42	0.00014	hh,sli,Diap1,Sin3A,Vang,tai,Stam,dysf,slbo,

Biological Process	tissue migration	10	4.18	0.00022	hh,sli,Diap1,Sin3A,Vang,tai,Stam,dysf,slbo,
Biological Process	ameboidal-type cell migration	10	3.99	0.00031	hh,sli,Diap1,Sin3A,Vang,tai,Stam,dysf,slbo,
Biological Process	open tracheal system development	14	3.66	4.9E-05	foi,dpp,hh,sli,lama,trh,rho,sano,noc,Stam,dysf, dmt,trh,tpr,
Biological Process	larval development	10	3.66	0.00059	dpp,hh,srp,Oatp74D,Sin3A,rho,Cht2,prc,bip1,ocm
Biological Process	respiratory system development	14	3.53	7.1E-05	foi,dpp,hh,sli,lama,trh,rho,sano,noc,Stam,dysf, dmt,trh,tpr,
Biological Process	chromatin remodeling	11	3.46	0.0005	Pc,ph-p,tara,escl,Mi-2,Nup153,His2B:CG33870, Su(var)2-HP2,tpr,aub,Hel25E,
Biological Process	cell migration	14	3.24	0.00017	foi,dpp,hh,pgc,sli,srp,Diap1,Sin3A,Vang,tai,Stam, robo2,dysf,slbo,
Biological Process	cell fate commitment	15	3.15	0.00013	cas,dpp,hh,ro,srp,trh,Brd,rho,Tom,E(spl)m8-HLH,daw,noc,robo2,Hey,ocm,
Biological Process	regulation of cell differentiation	14	3.12	0.00024	cas,dpp,hh,Lrrk,srp,bam,Nup154,E(spl)mdelta-HLH,E(spl)m8-HLH,daw,Vang,nerfin-1,Hey,He,
Biological Process	chromatin organization	13	3	0.00058	Pc,ph-p,tara,escl,pea,Mi-2,Nup153,His2B: CG33870,Su(var)2-HP2,tpr,ocm,aub,Hel25E
Biological Process	negative regulation of gene expression	15	2.94	0.00027	Achl,Pc,dpp,pgc,ph-p,escl,bam,Diap1,rho, E(spl)m8-HLH,SmydA-6,noc,Su(var)2-HP2, aub,dnr1,
Biological Process	negative regulation of DNA-templated transcription	15	2.94	0.00027	cas,Pc,pgc,ph-p,escl,srp,E(spl)mdelta-HLH,mrt,Sin3A,Mi-2,ovo,noc,nerfin-1,Rbf2,tpr,
Biological Process	negative regulation of nucleic acid-templated transcription	15	2.94	0.00027	cas,Pc,pgc,ph-p,escl,srp,E(spl)mdelta-HLH,mrt,Sin3A,Mi-2,ovo,noc,nerfin-1,Rbf2,tpr,
Biological Process	negative regulation of RNA biosynthetic process	15	2.94	0.00027	cas,Pc,pgc,ph-p,escl,srp,E(spl)mdelta-HLH,mrt,Sin3A,Mi-2,ovo,noc,nerfin-1,Rbf2,tpr,
Biological Process	negative regulation of RNA metabolic process	15	2.75	0.00052	cas,Pc,pgc,ph-p,escl,srp,E(spl)mdelta-HLH,mrt,Sin3A,Mi-2,ovo,noc,nerfin-1,Rbf2,tpr,
Biological Process	negative regulation of nucleobase-containing compound metabolic process	16	2.71	0.0004	cas,Pc,pgc,Rif1,ph-p,escl,srp,E(spl)mdelta-HLH,mrt,Sin3A,Mi-2,ovo,noc,nerfin-1,Rbf2,tpr,
Biological Process	negative regulation of cellular biosynthetic process	17	2.58	0.00047	dpp,hh,sli,ph-p,srp,bam,scw,nullo,trh,rho, lama,Tom,E(spl)m8-HLH,Mur11Da,Cp16 ,dunk,robo2,Femcoat,fln,aub,trh,Ndg,
Biological Process	anatomical structure formation involved in morphogenesis	22	2.58	6.7E-05	cas,Pc,pgc,ph-p,escl,srp,bam,E(spl)mdelta-HLH,mrt,Sin3A,Mi-2,ovo,noc,nerfin-1,Rbf2,tpr,dnr1,
Biological Process	tissue development	41	2.54	7.8E-08	foi,dpp,hh,sli,ro,Vajk3,ph-p,tara,lama,srp,scw, nullo,trh,Nup154,Tollo,Diap1,sha,bib,Sin3A,rho,Spn 88Eb,lama,E(spl)m8-HLH,ovo,Mur11Da, Cp16,daw,sano,nocVang,tai,Col4a1,robo2,ken,dysf ,Femcoat,dmt,vkg,aub,slbo,EloA,
Biological Process	negative regulation of biosynthetic process	17	2.54	0.00053	Achl,cas,Pc,dpp,hh,pgc,Rif1,ph-p,escl,srp,bam, E(spl)mdelta-HLH,mrt,Diap1,Spn28Dc,Sin3A,Mi-2,rho,Spn88Eb,E(spl)m8-HLH,ovo,SmydA-6, noc,nerfin-1,Rbf2,Su(var)2-HP2,tpr,aub,dnr1, ken,dysf,Femcoat,dmt,vkg,aub,slbo,EloA,
Biological Process	epithelium development	38	2.54	1.6E-07	cas,Pc,pgc,ph-p,escl,srp,bam,E(spl)mdelta-HLH,mrt,Sin3A,Mi-2,ovo,noc,nerfin-1,Rbf2,tpr,dnr1,

Biological Process	negative regulation of macromolecule metabolic process	29	2.52	6.7E-06	Achl,cas,Pc,dpp,hh,pgc,Rif1,ph-p,escl,srp,bam,E(spl)mdelta-HLH,mrt,Diap1,Spn28Dc,Sin3A,Mi-2,rho,Spn88Eb,E(spl)m8-HLH,ovo,SmydA-6,noc,nerfin-1,Rbf2,Su(var)2-HP2,tpr,aub,dnr1,
Biological Process	tissue morphogenesis	24	2.51	4.7E-05	foi,dpp,hh,sli,ro,ph-p,srp,scw,nullo,trh,Tollo,Diap1,sha,rho,ovo,daw,sano,noc,Vang,Col4a1,dysf,dmt,vkg,EloA,
Biological Process	negative regulation of nitrogen compound metabolic process	22	2.49	0.00011	cas,Pc,hh,pgc,Rif1,ph-p,escl,srp,bam, E(spl)mdelta-HLH,mrt,Diap1,Spn28Dc,Sin3A,Mi-2,Spn88Eb,ovo,noc,nerfin-1,Rbf2,tpr,dnr1,
Biological Process	morphogenesis of an epithelium	23	2.48	8.1E-05	foi,dpp,hh,ro,ph-p,srp,scw,nullo,trh, Tollo,Diap1,sha,rho,ovo,daw,sano,noc,Vang, Col4a1,dysf,dmt,vkg,EloA,
Biological Process	negative regulation of metabolic process	30	2.41	1.2E-05	Achl,cas,Pc,dpp,hh,pgc,Rif1,ph-p,escl,srp,bam, E(spl)mdelta-HLH,mrt,Diap1,Spn28Dc,Sin3A,Mi-2,rho,Spn88Eb,E(spl)m8-HLH,ovo,SmydA-6, daw,noc,nerfin-1,Rbf2,Su(var)2-HP2,tpr,aub,dnr1
Biological Process	regulation of transcription by RNA polymerase II	34	2.38	3.5E-06	fd3F,cas,Pc,ro,ph-p,escl,dmrt11E,srp, trh,Rcd5,E(spl)mdelta-HLH,Lime,rgr,Sin3A,Mi-2,ebd1,E(spl)m8-HLH,pre-lola-G,ovo,tai,Nup153, nerfin-1,fd59A,Rbf2,cato,Hey,ken,dysf,tpr, hang,Klf15,ocm,Opbp,slbo
Biological Process	multicellular organism development	81	2.37	2.3E-14	TwdlK,foi,cas,TwdlH,Pc,dpp,hh,sli,ro,Vajk3,Rif1,Atg17,ph-p,tara,lama,TwdlE,srp,bam,Cpr49Af,scw,nullo,trh,Nup154,E(spl)mdelta-HLH,Tollo,Diap1,Cp19, TwdlS,sha,Dcp-1,pea,Oatp74D,Sin3A,Prat, rho,Cpr72Ea,lama,Tom,E(spl)m8-HLH, Cpr65Ec, ovo,Cht2,TwdlC,Cp16,y,wcd,sano,daw,noc,Vang,spdo,Ziz,Col4a1,TwdlN,Stam,Cpr78Ca,dunk,TwdlX,nerfin-1,robo2,cato,Hey,ken,ytr,dysf,prc, Klf15,bip1,dmt,TwdlJ,Cpr78E,TwdlO,vkg,ocm,Cpr78Cc,TwdlR,aub,trh,tpr,EloA,Ndg,
Biological Process	developmental process	100	2.34	6.1E-18	TwdlK,fd3F,foi,cas,TwdlH,Pc,dpp,hh,pgc,sli,ro,Vajk3,Rif1,Atg17,ph-p,tara,dmrt11E,lama,TwdlE, srp,bam,Cpr49Af,scw,nullo,trh,Nup154,E(spl)mdelta-HLH,Tollo,Brd,Diap1,Cp19,TwdlS,sha,bib,Dcp-1,pea,Oatp74D,Sin3A,Mi-2,ebd1,Prat,rho, Cpr72Ea,Spn88Eb,lama,Tom,E(spl)m8-HLH, Cpr65Ec,ovo,TwdlC,Cht2,Mur11Da,Cp16,y,wcd,sano,daw,Yp1,noc,Vang,spdo,tai,Ziz,Col4a1,TwdlN,Stam,Cpr78Ca,dunk,TwdlX,nerfin-1,robo2,fd59A, Rbf2,cato,Hey,ken,ytr,dysf,tpr,Mos,Femcoat,prc,Klf15,bip1,dmt,TwdlJ,Cpr78E,Hip14,fln,TwdlO,vkg,ocm,Cpr78Cc,TwdlR,aub,trh,tpr,slbo,EloA,Ndg
Biological Process	animal organ morphogenesis	27	2.33	5.7E-05	foi,dpp,hh,sli,ro,Atg17,lama,srp,scw,trh,E(spl)mdelta-HLH,Diap1,sha,rho,Tom,E(spl)m8-HLH,ovo, daw,sano,noc,Vang,spdo,Col4a1,robo2,dysf,prc, EloA,
Biological Process	tube development	26	2.33	8.3E-05	foi,dpp,hh,sli,Vajk3,tara,srp,scw,trh,Nup154,Diap1,sha,Sin3A,rho,lama,ovo,daw,noc,Vang,Col4a1,robo2,ken,dysf,dmt,vkg,EloA,

Biological Process	anatomical structure development	94	2.31	2.3E-16	fd3F,TwdlK,foi,cas,TwdlH,Pc,dpp,hh,pgc,sli,ro,Vajk3,Rif1,Atg17,ph-p,tara,lama,TwdlE,srp,bam, Cpr49Af,scw,nullo,trh,Nup154,E(spl)mdelta-HLH, Tollo,Diap1,Cp19,TwdlS,sha,bib,Dcp-1,pea, Oatp74D,Sin3A,ebd1,Prat,rho,Cpr72Ea,Spn88Eb, lama,Tom,E(spl)m8-HLH,Cpr65Ec,ovo,Cht2, TwdlC,Mur11Da,Cp16,y,wcd,sano,daw,noc,Vang,spdo,tai,Ziz,Col4a1,TwdlN,Stam,Cpr78Ca,dunk,TwdlX,nerfin-1,robo2,fd59A,cato,Hey,ken,ytr,dysf, tpr,Mos,Femcoat,prc,Klf15,bip1,dmt,TwdlJ,Cpr78E,fln,TwdlO,vkg,ocm,Cpr78Cc,TwdlR,aub,trh,tpr,slbo, EloA,Ndg,
Biological Process	animal organ development	43	2.3	3.4E-07	foi,cas,Pc,dpp,hh,sli,ro,Vajk3,Atg17,tara,lama,srp,scw,trh,Nup154,E(spl)mdelta-HLH,Diap1,sha,pea, Sin3A,rho,lama,Tom,E(spl)m8-HLH,ovo,daw,sano,noc,Vang,spdo,Ziz,Col4a1,robo2,cato,ken,ytr,dysf,prc,bip1,dmt,vkg,ocm,EloA,
Biological Process	developmental process involved in reproduction	32	2.22	2.9E-05	foi,dpp,hh,pgc,sli,ph-p,dmrt11E,bam,scw, Nup154,Diap1,Dcp-1,pea,Mi-2,Prat,lama,ovo, Mur11Da,Cp16,wcd,Yp1,Vang,tai,Col4a1,robo2,ken,tpr,Mos,Femcoat,Hip14,aub,slbo
Biological Process	anatomical structure morphogenesis	52	2.21	7.6E-08	fd3F,cas,Pc,dpp,hh,pgc,sli,ro,Atg17,ph-p,lama, srp,bam,trh,Nup154,E(spl)mdelta-HLH,Tollo, Brd, Diap1,sha,Dcp-1,pea,Prat,rho,lama,Tom, E(spl)m8-HLH,ovo,Mur11Da,Cp16, wcd,daw,noc,Vang,tai, Col4a1,Stam,nerfin-1, robo2,fd59A, Rbf2, cato, Hey,ytr,tpr,Mos,Femcoat,Klf15,fln,ocm,aub,slbo,
Biological Process	positive regulation of cellular metabolic process	27	2.15	0.00021	fd3F,cas,Pc,pgc,ro,ph-p,escl,dmrt11E,srp,trh, Rcd5,E(spl)mdelta-HLH,mrt, Lime,rgr,Sin3A,Mi-2, ebd1,E(spl)m8-HLH,pre-lola-G,ovo,noc,tai, Nup153,nerfin-1,fd59A,Rbf2,cato,Hey,ken,dysf, tpr,hang,Klf15,ocm,Lst8,Opbp,Polr3A,slbo,
Biological Process	regulation of DNA-templated transcription	39	2.15	6.7E-06	fd3F,cas,Pc,pgc,ro,ph-p,escl,dmrt11E,srp,trh,Rcd5,E(spl)mdelta-HLH,mrt,Lime,rgr,Sin3A,Mi-2,ebd1,E(spl)m8-HLH,pre-lola-G,ovo,noc,tai,Nup153,nerfin-1,fd59A,Rbf2,cato,Hey,ken,dysf,tpr,hang,Klf15,ocm,Lst8,Opbp,Polr3A,slbo,
Biological Process	regulation of nucleic acid-templated transcription	39	2.15	6.7E-06	fd3F,cas,Pc,pgc,ro,ph-p,escl,dmrt11E,srp,trh,Rcd5,E(spl)mdelta-HLH,mrt,Lime,rgr,Sin3A,Mi-2,ebd1,E(spl)m8-HLH,pre-lola-G,ovo,noc,tai,Nup153,nerfin-1,fd59A,Rbf2,cato,Hey,ken,dysf,tpr,hang,Klf15,ocm,Lst8,Opbp,Polr3A,slbo,
Biological Process	regulation of RNA biosynthetic process	39	2.15	6.7E-06	cas,dpp,ro,Atg17,srp,scw,trh,Rcd5,PEK,Dcp-1,Tab2,rgr,Sin3A,ebd1,rho,ovo,daw,Hayan,tai,Nup153,dysf,tpr,Klf15,aub,Lst8,Polr3A,slbo,
Biological Process	system development	46	2.14	1.1E-06	foi,cas,Pc,dpp,hh,sli,ro,Atg17,ph-p,lama, srp,scw,trh,Nup154,E(spl)mdelta-HLH, Tollo, sha,Dcp-1,pea,rho,E(spl)m8-HLH,ovo,wcd,daw, sano,noc,Vang,spdo,Ziz,Col4a1,Stam,nerfin-1, robo2,cato,Hey,ken,ytr,dysf,prc,Klf15,bip1,dmt,vkg, ocm,trh,tpr,

Biological Process	multicellular organismal process	112	2.09	5.1E-17	TwdlK,foi,cas,Gr59b,TwdlH,trh,Pc,dpp,hh,pgc,sli,ro, Vajk3,Rif1,Atg17,inaD,Gr89a,ph-p,ETH,Mec2,tara,dmrt11E,lama,TwdlE,srp,bam,Cpr49Af,scw,nullo,trh,Or82a,Nup154,E(spl)mdelta-HLH,Sans,Tollo,nAChRbeta3,MRP,Diap1,Cp19,TwdlS,sha,mthl6,Dcp-1,pea,Oatp74D,Sin3A,Mi-2,Prat,rho,Cpr72Ea,lama,Tom,E(spl)m8-HLH, Cpr65Ec,ovo,TwdlC,Cht2,Mur11Da,Cp16,y,Nsun2, wcd,sano,daw,noc,Gr28b,Vang,spdo,tai,Ziz,Col4a1, TwdlN,Jhe,Stam,Cpr78Ca,dunk,Obp19c,TwdlX,nerfin-1,robo2,cato,Hey,Gba1a,ken,ytr, dysf,tpr,Mos,hang,Femcoat,prc,Klf15,bip1,dmt,TwdlJ,Cpr78E,Hip14,fln,TwdlO,vkg,ocm,Cpr78Cc,TwdlR,Or1a,aub,trh,tpr,slbo,lovit,dnr1,EloA,Ndg,
Biological Process	cellular developmental process	53	2.09	2.3E-07	fd3F,cas,Pc,dpp,hh,pgc,sli,ro,Atg17,ph-p,lama, srp,bam,trh,Nup154,E(spl)mdelta-HLH,Tollo,Brd, Diap1,sha,Dcp-1,pea,Prat,rho,lama,Tom,E(spl)m8-HLH,ovo,Mur11Da,Cp16,wcd,daw,noc,Vang,tai,Col4a1,Stam,nerfin-1,robo2,fd59A,Rbf2,cato,Hey, ytr,tpr,Mos,Femcoat,Klf15,fln,ocm,aub,slbo,Ndg,
Biological Process	cell differentiation	52	2.07	6.3E-07	fd3F,TwdlK,foi,dpp,hh,sli,ro,Atg17,ph-p,lama,srp,bam,scw,nullo,trh,E(spl)mdelta-HLH,Tollo,Diap1,TwdlS,sha,rho,lama,Tom,E(spl)m8-HLH,ovo,Mur11Da,Cp16,sano,daw,noc, Vang,spdo,Col4a1,TwdlN,dunk,TwdlX,nerfin-1, robo2,fd59A,dysf,Femcoat,prc,dmt,TwdlJ,fln,TwdlO, vkg,TwdlR,aub,trh,EloA,Ndg,
Biological Process	positive regulation of metabolic process	30	2.04	0.00022	cas,dpp,ro,Atg17,tara,srp,bam,scw,trh,Rcd5,PEK,Diap1,Dcp-1,Tab2,rgr,Sin3A,ebd1,rho,ovo,daw, Hayan,tai,Nup153,dysf,tpr,Klf15,aub,Lst8,Polr3A, slbo
Biological Process	positive regulation of macromolecule metabolic process	27	2.04	0.00053	cas,dpp,ro,Atg17,tara,srp,bam,scw,trh,Rcd5,Diap1, Tab2,rgr,Sin3A,ebd1,rho,ovo,daw,tai,Nup153,dysf,tpr,Klf15,aub,Lst8,Polr3A,slbo,
Biological Process	regulation of RNA metabolic process	42	2.03	1.3E-05	fd3F,cas,Pc,pgc,ro,ph-p,escl,dmrt11E, srp,bam,trh,Rcd5,E(spl)mdelta-HLH,Tollo,mrt,Lime,Tab2,rgr,Sin3A,Mi-2,ebd1, E(spl)m8-HLH,pre-lola-G,ovo,noc,tai,Nup153, nerfin-1,fd59A,Rbf2,cato,Hey,ken,dysf,tpr, hang,Klf15,ocm,aub,Lst8,Opbp,Polr3A,slbo,dnr1,
Biological Process	regulation of cellular biosynthetic process	44	2.03	7.3E-06	fd3F,cas,Pc,pgc,ro,ph-p,escl,dmrt11E, srp,bam,trh,Rcd5,E(spl)mdelta-HLH,Tollo,mrt, Lime,rgr,Sin3A,Mi-2,ebd1,E(spl)m8-HLH,pre-lola-G,ovo,noc,tai,Nup153,nerfin-1,fd59A, Rbf2,cato,ken,dysf,tpr,hang,Klf15,ocm,aub,Lst8, Opbp,Polr3A,slbo,Hel25E,
Biological Process	regulation of gene expression	53	2.02	6.4E-07	Achl,fd3F,cas,Pc,dpp,hh,pgc,ro,ph-p,tara,escl,dmrt11E,srp,bam,trh,Rcd5,E(spl)mdelta-HLH,mrt,Diap1,Lime,pea,Tab2,rgr,Sin3A,Mi-2,ebd1,rho,E(spl)m8-HLH,pre-lola-G,ovo,SmydA-6,noc,tai,Nup153,nerfin-1,fd59A,Rbf2,Su(var)2-HP2,cato,Hey,ken,dysf,tpr,hang,Klf15,ocm,aub,Lst8,Opbp,Polr3A,slbo,dnr1,Hel25E,
Biological Process	regulation of macromolecule biosynthetic process	42	2.02	1.4E-05	fd3F,cas,Pc,pgc,ro,ph-p,escl,dmrt11E,srp,bam,trh,Rcd5,E(spl)mdelta-HLH,Tollo,mrt,Lime,rgr,Sin3A,Mi-2,ebd1,E(spl)m8-HLH,pre-lola-G,ovo,noc,tai,Nup153,nerfin-1,fd59A,Rbf2,cato,ken,dysf,tpr,hang,Klf15,ocm,aub, Lst8,Opbp,Polr3A,slbo,Hel25E,

Biological Process	regulation of biosynthetic process	44	2.01	8.3E-06	fd3F,cas,Pc,pgc,ro,ph-p,escl,dmrt11E,srp,bam,trh,Rcd5,E(spl)mdelta-HLH,Tollo,mrt,Lime,Tab2,rgr,Sin3A,Mi-2,ebd1,E(spl)m8-HLH,pre-lola-G,ovo,noc,tai,Nup153,nerfin-1,fd59A,Rbf2,cato,Hey,ken,dysf,tpr,hang,Klf15,ocm,aub,Lst8,Opbp,Polr3A,slbo,dnr1,
Biological Process	regulation of nucleobase-containing compound metabolic process	43	1.97	2.1E-05	fd3F,cas,Pc,pgc,ro,Rif1,ph-p,escl,dmrt11E,srp,trh,Rcd5,E(spl)mdelta-HLH,mrt,Lime,pea,rgr,Sin3A,Mi-2,ebd1,E(spl)m8-HLH,pre-lola-G,ovo,noc,tai,Nup153,nerfin-1,fd59A,Rbf2,cato,Hey,ken,dysf,tpr,hang,Klf15,ocm,aub,Lst8,Opbp,Polr3A,slbo,Hel25E,
Biological Process	regulation of nitrogen compound metabolic process	55	1.9	3E-06	fd3F,cas,Pc,dpp,hh,pgc,ro,Rif1,Atg17,ph-p,escl,dmrt11E,srp,bam,scw,trh,Rcd5,E(spl)mdelta-HLH,Tollo,mrt,Diap1,Lime,Spn28Dc,pea,Tab2,rgr,Sin3A,Mi-2,ebd1,Spn88Eb,E(spl)m8-HLH,pre-lola-G,ovo,daw,noc,tai,Nup153,nerfin-1,fd59A,Rbf2,cato,Hey,ken,dysf,tpr,hang,Klf15,ocm,aub,Lst8,Opbp,Polr3A,slbo,dnr1,Hel25E,
Biological Process	regulation of cellular metabolic process	56	1.87	4.3E-06	Achl,fd3F,cas,Pc,dpp,hh,pgc,ro,Rif1,Atg17,ph-p,tara,escl,dmrt11E,srp,bam,scw,trh,Rcd5,E(spl)mdelta-HLH,Tollo,mrt,Diap1,Lime, Spn28Dc, pea,Tab2,rgr,Sin3A,Mi-2,ebd1, rho, Spn88Eb,E(spl)m8-HLH,pre-lola-G,ovo, SmydA-6,daw,noc,tai,Nup153,nerfin-1,fd59A, Rbf2, Su(var)2-HP2,cato,Hey,ken,dysf,tpr,hang, Klf15, ocm,aub,Lst8,Opbp,Polr3A,slbo,dnr1,Hel25E,
Biological Process	regulation of macromolecule metabolic process	60	1.87	1.3E-06	fd3F,cas,Pc,dpp,pgc,ro,Rif1,Atg17,ph-p,escl,dmrt11E,srp,bam,scw,trh,Rcd5,PEK,E(spl)mdelta-HLH,Tollo,mrt,Dcp-1,Lime,Spn28Dc,pea,Tab2,rgr,Sin3A,Mi-2,ebd1,rho,E(spl)m8-HLH,pre-lola-G,ovo,daw,noc,Hayan,tai,Nup153,nerfin-1,fd59A,Rbf2,cato,Hey,ken,dysf,tpr,hang,Klf15,ocm,aub,Lst8,Opbp,Polr3A,slbo,dnr1,Hel25E,
Biological Process	reproductive process	36	1.87	0.00029	foi,dpp,hh,pgc,sli,Fancd2,ph-p,dmrt11E,srp,bam,scw,Nup154,Diap1,Dcp-1,pea,Mi-2,Prat,lama,ovo,Mur11Da,Cp16,y, wcd,Yp1,Vang,tai,Col4a1,Jhe,robo2,ken,tpr,Mos,Femcoat,Hip14,aub,slbo,
Biological Process	negative regulation of cellular process	37	1.85	0.00035	cas,Pc,Mcm2,hh,pgc,Lrrk,Rif1,Fancd2,ph-p,escl,lama,srp,bam,E(spl)mdelta-HLH,mrt,Brd,Diap1,Dcp-1,Spn28Dc,Sin3A,Mi-2,rho,Tom,ovo,daw,noc,spdo,nerfin-1,Rbf2,ken,tpr,Mos,hang,He,dmt,aub,dnr1,
Biological Process	regulation of metabolic process	63	1.81	2.7E-06	Achl,fd3F,cas,Pc,dpp,hh,pgc,ro,Rif1,Atg17,ph-p,tara,escl,dmrt11E,srp,bam,scw,trh,Rcd5,PEK, E(spl)mdelta-HLH,Tollo,mrt,Diap1,Dcp-1, Lime,Spn28Dc,pea,Tab2,rgr,Sin3A,Mi-2,ebd1, rho,Spn88Eb,E(spl)m8-HLH,pre-lola-G, ovo,SmydA-6,daw,noc,Hayan,tai,Nup153, nerfin-1,fd59A,Rbf2,Su(var)2-HP2,cato, Hey,ken,dysf,tpr,hang,Klf15,ocm,aub,Lst8,Opbp, Polr3A,slbo,dnr1,Hel25E,
Biological Process	regulation of primary metabolic process	53	1.8	2.4E-05	Achl,fd3F,cas,Pc,dpp,hh,pgc,ro,ph-p,tara,escl,dmrt11E,srp,bam,trh,Rcd5,E(spl)mdelta-HLH,mrt,Diap1,Lime,pea,Tab2,rgr,Sin3A,Mi-2,ebd1,rho,E(spl)m8-HLH,pre-lola-G,ovo,SmydA-6,noc,tai,Nup153,nerfin-1,fd59A,Rbf2,Su(var)2-HP2,cato,Hey,ken,dysf,tpr,hang,Klf15,ocm,aub,Lst8,Opbp,Polr3A,slbo,dnr1,Hel25E,

Biological Process	negative regulation of biological process	43	1.8	0.00019	foi,cas,Pc,dpp,hh,sli,ro,Vajk3,Atg17,tara,lama,srp,scw,trh,Nup154,E(spl)mdelta-HLH,Diap1,sha, pea,Sin3A,rho,lama,Tom,E(spl)m8-HLH,ovo, daw,sano,noc,Vang,spdo,Ziz,Col4a1,robo2,cato, ken,ytr,dysf,prc,bip1,dmt,vkg,ocm,EloA,
Biological Process	response to stimulus	66	1.62	3.8E-05	Pc,dpp,Mcm2,hh,Ir51b,Ir7d,santa-maria,Lrrk, sli,Atg17,inaD,Gr89a,Fancd2,ph-p,ETH,lama, Gss2,scw,Or82a,PEK,kst,Tollo,nAChRbeta3,MRP,TotA,TotX,mthl6,Dcp-1,Spn28Dc,Tab2,Oatp74D, rho,TotC,Spn88Eb,Zip89B,Ir7b,Cht2,daw,Fst,Drsl4, Gr28b,Vang,Hayan,tai,p38c,Col4a1,slx1,Jhe,Stam, Karl,nerfin-1,robo2,Rgl,Sid,Gba1a,Ir7f,tpr, Mos,hang,Klf15,Ir7e,He,dmt,Or1a,aub,Lst8,
Biological Process	regulation of biological process	98	1.51	3.4E-06	Achl,fd3F,cas,Pc,dpp,Mcm2,hh,pgc,santa-maria,Lrrk,sli,ro,Rif1,Atg17,inaD,Fancd2,ph-p,ETH,tara,dmrt11E,escl,lama,srp,bam,scw,nullo,trh,Rcd5,Nup154,PEK,E(spl)mdelta-HLH,Tollo,nAChRbeta3,mrt,Brd,Diap1,mthl6,Dcp-1,Lime,Spn28Dc,pea,Tab2,rgr,CG1139,Oatp74D,Sin3A,Mi-2,ebd1,rho,Kmn1,Spn88Eb, Tom,E(spl)m8-HLH,pre-lola-G,ovo,y,SmydA-6, daw,noc,Gr28b,Vang,Hayan,spdo,tai,p38c,Ziz,Col4a1,Nup153,Stam,nerfin-1,robo2,fd59A, Rgl,Rbf2,Su(var)2-HP2,cato,IFT54,Hey,ken,dysf, tpr,Mos,hang,Klf15,He,dmt,Hip14,ocm,tank,aub, trh,Lst8,Opbp,Polr3A,slbo,dnr1,Hel25E,Ndg,
Biological Process	regulation of cellular process	89	1.5	1.7E-05	fd3F,cas,Pc,dpp,Mcm2,hh,pgc,santa-maria, Lrrk,sli,ro,Rif1,Atg17,inaD,Fancd2,ph-p,ETH, escl,dmrt11E,lama,srp,bam,scw,trh,Rcd5,Nup154,PEK,E(spl)mdelta-HLH,Tollo,nAChRbeta3, mrt,Brd,Diap1,mthl6,Dcp-1,Lime,Spn28Dc,pea, Tab2,rgr,Oatp74D,Sin3A,Mi-2,ebd1,rho,Kmn1, Spn88Eb,Tom,E(spl)m8-HLH,pre-lola-G,ovo, daw,noc,Gr28b,Vang, Hayan,spdo,tai,p38c,Ziz, Col4a1,Nup153,Stam,nerfin-1,robo2,fd59A, Rgl,Rbf2,IFT54,cato,Hey,ken, dysf,tpr, Mos,hang,Klf15,He,dmt,Hip14,ocm,aub, Lst8,Opbp,Polr3A,slbo,dnr1,Hel25E,Ndg,
Biological Process	biological regulation	102	1.42	3.4E-05	Achl,fd3F,foi,cas,Pc,dpp,Mcm2,hh,pgc,santa-maria,Lrrk,sli,ro,Rif1,Atg17,inaD,Fancd2,ph-p,ETH,tara,dmrt11E,escl,lama,srp,bam,scw,nullo,trh,Rcd5,Nup154,PEK,E(spl)mdelta-HLH,Tollo, nAChRbeta3,mrt,Brd,Diap1,mthl6,Dcp-1,Lime, Spn28Dc,pea,Tab2,rgr,CG1139,Oatp74D,Sin3A,Mi-2,ebd1,rho,Kmn1,Spn88Eb, Tom,E(spl)m8-HLH,pre-lola-G,ovo,y,SmydA-6, sano,daw,noc, Gr28b,Vang,Hayan,spdo,tai,p38c,Ziz,Col4a1,Nup153,Jhe,Stam,nerfin-1,robo2, fd59A,Rgl,Rbf2, Su(var)2-HP2,cato,IFT54, Hey,Gba1a,ken,dysf,tpr, Mos,hang,Klf15,He,dmt,Hip14,ocm,tank,aub,trh, Lst8,Opbp,Polr3A,slbo,dnr1,Hel25E,Ndg,
Molecular Function	extracellular matrix structural constituent	9	16.98	1.5E-08	Ppn,Mur11Da,Muc30E,Muc55B,Col4a1,prc,vkg,Muc68E,Ndg,
Molecular Function	structural constituent of chitin-based cuticle	17	7.2	9E-10	TwdlK,TwdlH,Vajk3,TwdlE,Cpr49Af,TwdlS,Cpr72Ea,Cpr65Ec,TwdlC,TwdlN,Cpr78Ca,TwdlX,TwdlJ,Cpr78E,TwdlO,Cpr78Cc,TwdlR
Molecular Function	RNA helicase activity	6	7.18	0.0003	pit,kz,pea,CG9630,CG8611,Hel25E
Molecular Function	structural constituent of cuticle	17	7.15	9.9E-10	TwdlK,TwdlH,Vajk3,TwdlE,Cpr49Af,TwdlS,Cpr72Ea,Cpr65Ec,TwdlC,TwdlN,Cpr78Ca,TwdlX,TwdlJ,Cpr78E,TwdlO,Cpr78Cc,TwdlR,

Molecular Function	structural molecule activity	32	4.23	1.6E-11	TwdlK,TwdlH,Ppn,Vajk3,TwdlE,Cpr49Af,Nup154,TwdlS,Cpr72Ea,Cpr65Ec,TwdlC,Mur11Da,Muc30E,Muc55B,Col4a1,TwdlN,Nup153,Cpr78Ca,TwdlX,His2B:CG33870,tpr,Femcoat,prc,TwdlJ,Cpr78E,fln,TwdlO,vkg,Cpr78Cc,TwdlR,Muc68E,Ndg,
Molecular Function	RNA polymerase II transcription regulatory region sequence-specific DNA binding	21	2.65	6.9E-05	fd3F,cas,ro,dmrt11E,srp,trh,E(spl)mdelta-HLH,E(spl)m8-HLH,ovo,nerfin-1,fd59A,Rbf2, cato,Hey,ken,dysf,Klf15,Pbp45,ocm,Opbp,slbo
Molecular Function	DNA-binding transcription factor activity, RNA polymerase II-specific	21	2.64	7.1E-05	fd3F,cas,ro,dmrt11E,srp,trh,E(spl)mdelta-HLH,E(spl)m8-HLH,ovo,nerfin-1,fd59A,Rbf2, cato,Hey,ken,dysf,Klf15,Pbp45,ocm,Opbp,slbo
Molecular Function	DNA-binding transcription factor activity	23	2.57	4.7E-05	fd3F,cas,ro,dmrt11E,srp,trh,E(spl)mdelta-HLH, Lime,rgr,E(spl)m8-HLH,ovo,nerfin-1,fd59A, Rbf2, cato,Hey,ken,dysf,hang,Klf15,ocm,Opbp,slbo,
Molecular Function	sequence-specific DNA binding	26	2.55	1.7E-05	fd3F,cas,Pc,ro,ph-p,dmrt11E,srp,trh,E(spl)mdelta-HLH,mrt,Lime,E(spl)m8-HLH,ovo,nerfin-1, fd59A,Rbf2,cato,Hey,ken,dysf,hang,Klf15,Pbp45, ocm,Opbp,slbo,
Molecular Function	transcription cis-regulatory region binding	22	2.52	9.1E-05	fd3F,cas,ro,dmrt11E,srp,trh,E(spl)mdelta-HLH, mrt,E(spl)m8-HLH,ovo,nerfin-1,fd59A, Rbf2,cato, Hey,ken,dysf,Klf15,Pbp45,ocm,Opbp,slbo,
Molecular Function	transcription regulatory region nucleic acid binding	22	2.52	9.3E-05	fd3F,cas,ro,dmrt11E,srp,trh,E(spl)mdelta-HLH, mrt,E(spl)m8-HLH,ovo,nerfin-1,fd59A,Rbf2, cato,Hey,ken,dysf,Klf15,Pbp45,ocm,Opbp,slbo,
Molecular Function	sequence-specific double-stranded DNA binding	22	2.41	0.00024	fd3F,cas,ro,dmrt11E,srp,trh,E(spl)mdelta-HLH, mrt,E(spl)m8-HLH,ovo,nerfin-1,fd59A, Rbf2,cato, Hey,ken,dysf,Klf15,Pbp45,ocm,Opbp,slbo,
Molecular Function	DNA binding	34	2.23	1.5E-05	fd3F,cas,Pc,Mcm2,ro,ph-p,dmrt11E,srp,trh, E(spl)mdelta-HLH,mrt,Lime,Mi-2,ebd1,E(spl)m8-HLH,ovo,Nup153,nerfin-1,fd59A,His2B:CG33870, Rbf2,cato,Su(var)2-HP2,Hey,ken,dysf,tpr,hang, Klf15,Pbp45,ocm,Opbp,Polr3A,slbo,
Molecular Function	transcription regulator activity	26	2.21	0.00022	fd3F,cas,ro,tara,dmrt11E,srp,trh,E(spl)mdelta-HLH,Lime,rgr,Sin3A,E(spl)m8-HLH,ovo,tai,nerfin-1,fd59A,Rbf2,cato,Hey,ken,dysf,hang,Klf15,ocm,Opbp,slbo,
Molecular Function	nucleic acid binding	53	1.92	3.2E-06	Achl,fd3F,cas,Pc,Mcm2,kz,pit,ro,ph-p, dmrt11E, eIF2D,srp,bam,trh,Rcd5,E(spl)mdelta-HLH,mrt, Pus7,Mat89Ba,Lime,pea,Mi-2,ebd1,E(spl)m8-HLH,ovo,Nsun2,Regnase-1,l(1)G0020, Nup153,nerfin-1,fd59A,His2B:CG33870, Rbf2,Su(var)2-HP2,Sid,cato,Hey,ken,ytr, dysf,tpr,hang,Klf15,Pbp45,ocm,CG9630,aub,Opbp, Ref1,Polr3A,CG8611,slbo,Hel25E,
Molecular Function	binding	116	1.32	0.00018	Achl,fd3F,cas,trh,Pc,dpp,Mcm2,hh,CG17904,Lrrk,pit,kz,sli,ro,Vajk3,Rif1,Cyp316a1,Atg17,inaD,Fancd2, ph-p,ETH,Mec2,dmrt11E,escl,eIF2D, lama,srp,bam,Gss2,scw,trh,dnk,Or82a,lectin-24Db,Rcd5,Nup154,PEK,E(spl)mdelta-HLH,Cftr,Sans, kst,mrt,Brd,MRP,Diap1, Pus7,Mat89Ba,Dcp-1, Lime,pea,Tab2,Sin3A,Mi-2,ebd1,Tom,E(spl)m8-HLH,Kif19A,ovo,Cht2, Nsun2,SmydA-6,daw, Regnase-1,noc, Vang,spdo,l(1)G0020,tai, p38c,Arl6,Nup153, slx1,Jhe,Stam,Dip-B, Obp19c,nerfin-1,robo2, fd59A,His2B:CG33870, Rgl,Rbf2,Su(var)2-HP2,cato,IFT54,Sid,Hey,ken,ytr, dysf,tpr,Mos, hang,Sgsh,Klf15,bip1,Pbp45,ocm,thw,Or1a,CG9630,Coq8,aub,Muc68E,Pex7,trh,Lst8,Opbp,Polr3A,Ref1,CG8611,slbo,dnr1,Hel25E,Ndg
Cellular Component	collagen type IV trimer	3	62.26	7.5E-05	Col4a1,prc,vkg,

Cellular Component	basement membrane collagen trimer	3	62.26	7.5E-05	Col4a1,prc,vkg,
Cellular Component	network-forming collagen trimer	3	62.26	7.5E-05	Col4a1,prc,vkg,
Cellular Component	collagen network	3	62.26	7.5E-05	Col4a1,prc,vkg,
Cellular Component	complex of collagen trimers	3	62.26	7.5E-05	Col4a1,prc,vkg,
Cellular Component	collagen trimer	3	46.69	0.00013	Col4a1,prc,vkg,
Cellular Component	basement membrane	6	17.79	3.3E-06	Ppn,lama,Col4a1,prc,vkg,Ndg,
Cellular Component	collagen-containing extracellular matrix	6	8.69	0.00012	Ppn,lama,Col4a1,prc,vkg,Ndg,
Cellular Component	extracellular matrix	27	6.16	2.4E-13	TwdlK,TwdlH,Ppn,Vajk3,lama,TwdlE,Cpr49Af,Cpr76Bb,Cpr72Ea,Cpr65Ec,TwdlC,Mur11Da,Muc30E,Muc55B,Col4a1,TwdlN,Cpr78Ca,TwdlX,prc,TwdlJ,Cpr78E,TwdlO,vkg,Cpr78Cc,Muc68E,Sgs7,Lcp9,Ndg,
Cellular Component	external encapsulating structure	29	5.88	8.4E-14	TwdlK,TwdlH,Ppn,Vajk3,lama,TwdlE,Cpr49Af,Cp19,TwdlS,Cpr72Ea,Cpr65Ec,TwdlC,Mur11Da,Cp16,Muc30E,Muc55B,Col4a1,TwdlN,Cpr78Ca,TwdlX,prc,TwdlJ,Cpr78E,TwdlO,vkg,Cpr78Cc,TwdlR,Muc68E,Ndg,
Cellular Component	chitin-based extracellular matrix	9	4.24	0.0004	TwdlH,Vajk3,Cpr49Af,Cpr76Bb,Cpr72Ea,Cpr65Ec,Cpr78Ca,TwdlJ,Cpr78E,Cpr78Cc,Lcp9,
Cellular Component	nucleolus	12	3.23	0.00049	Pc,pit,kz,ph-p,Mat89Ba,Nsun2,wcd,l(1)G0020,tpr,Nopp140,l(3)72Dn,CG9630
Cellular Component	cell periphery	60	1.97	2.3E-07	TwdlK,foi,TwdlH,Ppn,hh,OSCP1,sli,Vajk3,inaD,Mec2,Osi2,lama,TwdlE,Cpr49Af,Or82a,lectin-24Db,Eglp3,kst,Tollo,nAChRbeta3,MRP,Cp19,TwdlS,sha,bib,Osi17,Oatp74D,Nepl20,Cpr72Ea,Zip89B,Cpr65Ec,TwdlC,Mur11Da,Cp16,ppk13,sano,Muc30E,Vang,Tsp42Eb,spdo,Tsp42El,Muc55B,Col4a1,TwdlN,Cpr78Ca,dunk,TwdlX,Rgl,prc,TwdlJ,Cpr78E,TwdlO,vkg,Osi3,Cpr78Cc,TwdlR,Or1a,Muc68E,Osi19,Ndg,
Cellular Component	cellular anatomical entity	198	1.33	4.7E-14	Achl,TwdlK,foi,cas,Gr59b,TwdlH,Pc,Ppn,Mcm2,dpp,hh,Ir51b,OSCP1,Ir7d,Cluap1,CG17904,pgc,santa-maria,Lrrk,pit,kz,sli,ro,Vajk3,Rif1,Atg17,inaD,Gr89a,Fancd2,ph-p,ETH,Mec2,tara,Osi2,dmrt11E,escl,eIF2D,lama,TwdlE,srp,bam,Gss2,Cpr49Af,scw,trh,nullo,dnk,Or82a,lectin-24Db,Eglp3, Rcd5,Nup154,PEK,E(spl)mdelta-HLH,Cftr, Sans,nAChRbeta3,kst,Tollo,mrt,MRP,Ugt302C1,Diap1,Pus7,TotA,Cp19,TotX,TwdlS,Mat89Ba,sha,bib,Dcp-1,mthl6,Lime,Osi17,Spn28Dc,pea,rgr, Sin3A,Oatp74D,Mi-2,NimC3,Nepl20,rho, Cpr72Ea,TotC,Kmn1,GstD7,Sfp79B,Spn88Eb,Zip89B,lama,Art8,Tom,Ir7b,E(spl)m8-HLH,Cpr65Ec, Kif19A,pre-lola-G,ovo,CG7988,Cht2,TwdlC, Mur11Da,Cp16,y,Nsun2,Sardh,wcd,ppk13,sano,daw,Yp1,Fst,Regnase-1,Drsl4,Muc30E,noc,Gr28b, Vang,Tsp42Eb,mst,spdo,l(1)G0020,Tsp42El,tai,p38c,Muc55B,Arl6,Col4a1,TwdlN,Nup153,slx1,Jhe,Ccdc58,Stam,Cpr78Ca,Dip-B,dunk,Karl,Obp19c, TwdlX,nerfin-1,robo2,His2B:CG33870,Rgl,Rbf2, Su(var)2-HP2,IFT54,Sid,cato,Hey,ken,Ir7f,ytr,dysf, tpr,Mos,hang,Femcoat,prc,Klf15,Ir7e,dmt,TwdlJ,Cpr78E,Hip14,fln,TwdlO,vkg,Nopp140,ocm,l(3)72Dn,Osi3,thw,Cpr78Cc,TwdlR,Or1a,CG9630,Coq8,tank,aub,Muc68E,Pex7,trh,tpr,Lst8,Opbp,Polr3A,Ref1,CG8611,Osi19,slbo,lovit,dnr1,Hel25E,EloA,Ndg
